# Comprehension and engagement in survey interviews with virtual agents

**DOI:** 10.3389/fpsyg.2015.01578

**Published:** 2015-10-20

**Authors:** Frederick G. Conrad, Michael F. Schober, Matt Jans, Rachel A. Orlowski, Daniel Nielsen, Rachel Levenstein

**Affiliations:** ^1^Michigan Program in Survey Methodology, Institute for Social Research, University of MichiganAnn Arbor, MI, USA; ^2^Joint Program in Survey Methodology, University of MarylandCollege Park, MD, USA; ^3^Department of Psychology, New School for Social ResearchNew York, NY, USA; ^4^Center for Health Policy Research, University of California at Los AngelesLos Angeles, CA, USA; ^5^Department of Epidemiology, School of Public Health, University of MichiganAnn Arbor, MI, USA; ^6^Department of Biostatistics, Center for Cancer Biostatistics, University of Michigan Medical SchoolAnn Arbor, MI, USA; ^7^University of Chicago Consortium on Chicago School Research, Urban Education Institute, University of ChicagoChicago, IL, USA

**Keywords:** virtual agent, survey interviewing, social signals, comprehension, dialog capability, facial animation

## Abstract

This study investigates how an onscreen virtual agent's dialog capability and facial animation affect survey respondents' comprehension and engagement in “face-to-face” interviews, using questions from US government surveys whose results have far-reaching impact on national policies. In the study, 73 laboratory participants were randomly assigned to respond in one of four interviewing conditions, in which the virtual agent had either high or low dialog capability (implemented through Wizard of Oz) and high or low facial animation, based on motion capture from a human interviewer. Respondents, whose faces were visible to the Wizard (and videorecorded) during the interviews, answered 12 questions about housing, employment, and purchases on the basis of fictional scenarios designed to allow measurement of comprehension accuracy, defined as the fit between responses and US government definitions. Respondents answered more accurately with the high-dialog-capability agents, requesting clarification more often particularly for ambiguous scenarios; and they generally treated the high-dialog-capability interviewers more socially, looking at the interviewer more and judging high-dialog-capability agents as more personal and less distant. Greater interviewer facial animation did not affect response accuracy, but it led to more displays of engagement—acknowledgments (verbal and visual) and smiles—and to the virtual interviewer's being rated as less natural. The pattern of results suggests that a virtual agent's dialog capability and facial animation differently affect survey respondents' experience of interviews, behavioral displays, and comprehension, and thus the accuracy of their responses. The pattern of results also suggests design considerations for building survey interviewing agents, which may differ depending on the kinds of survey questions (sensitive or not) that are asked.

## Introduction

An important source of knowledge about society is what people report in survey interviews that produce the data for official (government) statistics, e.g., population estimates on employment, health and crime. Data from such survey interviews, which provide essential input for policy decisions, are administered on a very large scale; for example, more than 60,000 US households per month are recruited to participate in the Current Population Survey, from which the US unemployment rate is calculated, and for the European Social Survey (ESS) in 2012, 54,600 standardized face-to-face interviews were carried out in 29 countries (Ferrin and Kriesi, 2014). Results from these interviews can have far-reaching consequences: even small changes in reported US unemployment rates, for example, can affect world financial markets, and results from the ESS make “a major contribution to the creation of effective social and economic policies in Europe” (Geoghegan-Quinn, 2012). So understanding what leads to accurate responses, and to participants' willingness to engage in such surveys, is societally important (Schober and Conrad, 2015).

Although survey interviews have traditionally been administered by humans either face-to-face or on the telephone, the landscape is changing: surveys are increasingly “self-administered” (that is, administered by automated systems, as in online surveys in a web browser possibly on a mobile device; Mavletova and Couper, 2014), and new human and automated modes are being explored (Conrad and Schober, 2008), e.g., videomediated interviewing (Anderson, 2008), text message surveys (Schober et al., 2015), and speech dialog system surveys (Bloom, 2008; Johnston et al., 2013). Exploring new ways of administering surveys is sensible given declining survey response rates and the growing expenses of carrying out human-administered interviews (see, e.g., Groves, 2011; Keeter, 2012; Massey and Tourangeau, 2013), but the task is complex: new interviewing methods will only be adopted if they lead to high quality data (accurate responses, and response and completion rates comparable to or better than those in other modes) and to respondents satisfied with their experience.

One new interviewing technology that has been proposed to promote high quality data—as measured by disclosure of sensitive information and (presumably more) honest responding—uses animated virtual humans to ask questions and capture responses (Lucas et al., 2014; see also DeVault et al., 2014; Gratch et al., 2014). The promise is that virtual interviewers can promote rapport and engagement with participants while simultaneously providing a feeling of safety and anonymity that is much more difficult to achieve with a human interviewer, and at the same time allowing users to display (and even learn to improve) the social cues they display in interaction with humans (Baur et al., 2013). And some of the findings are promising: Lucas et al. (2014) found that people in a semi-structured clinical health screening interview disclosed more sensitive information in open-ended responses to a virtual interviewer they believed was automated than to one that was clearly operated by a human. von der Pütten et al. (2011) found that a more talkative interviewing agent led students to reveal more personal information and to produce more words in answering some open-ended questions on love and relationships.

The evidence on how virtual interviewers might affect responses in surveys that produce social science and government data, on the other hand, is less promising with respect to disclosure. The one study thus far (Lind et al., 2013) focused on responses to questions about sensitive and potentially embarrassing topics (alcohol and drug use, sexual behavior) and questions about personal behaviors (exercise, religious attendance); such questions can lead at least some respondents to answer in ways that present themselves in a more positive light in survey interviews where human interviewers ask the questions compared to when a computer presents textual or spoken questions (Tourangeau and Smith, 1996; Turner et al., 1998; Kreuter et al., 2008). The finding was that automation did increase disclosure relative to a human interviewer, but only with the audio-only (no facial representation) interface; there were few if any differences in responses to the virtual interviewers relative to a human interviewer (Lind et al., 2013).

Here we explore how virtual interviewers affect answers to the kinds of questions about facts and behaviors (e.g., “How many bedrooms are there in your house?” “Last week did you do any work for pay?”) that are especially common in survey interviews that produce official statistics and that, in most cases, are not particularly threatening or embarrassing to answer. Because these questions generally concern non-sensitive, mundane topics, we are not focused on how virtual human interviewers might affect disclosure. Instead, we explore how and whether virtual human interviewers promote conscientious task performance—accurate survey responding, which depends on comprehending the questions in the way the survey designers intended—and respondent engagement in these particular kinds of interviews. In our experiment we varied two features (among the many other potentially manipulable features of a virtual survey interviewer, see Lind et al., 2013)—the interviewer's *dialog capability* and *facial animation—*and explored whether they have independent or compound effects.

### Background

The kinds of survey interviews we examine here have particular features that distinguish them from other kinds of interaction (Schaeffer, 1991; Houtkoop-Steenstra, 2000; Schober and Conrad, 2002), as well as from other kinds of interviews. The survey interview is an interactive situation in which (usually) the interviewer, as a representative of the survey designers (researchers), initiates the dialog and “drives” the interaction according to a script (Suchman and Jordan, 1990), asking the respondent questions (that usually specify the answer categories) about her opinions and behaviors.

This kind of standardized wording and administration procedure is intended to make responses comparable across interviews. In the most strictly standardized interviews, interviewers are required to ask questions exactly as scripted and use only “neutral probes” like “Let me repeat the question” or “Whatever it means to you” if respondents say anything that isn't an acceptable answer (e.g., something other than a response option included in the question), so as to ensure that all respondents receive the same stimulus and to avoid the possibility that interviewers will bias responses (Fowler and Mangione, 1990). This can lead to perverse interactions in which interviewers thwart respondents' efforts to understand what they are being asked by refusing to provide the clarification that respondents seek (Suchman and Jordan, 1990), and in which interviewers violate ordinary norms of conversation by failing to “ground” the meaning of utterances they themselves have produced (Schober and Conrad, 2002).

Analyses of these kinds of survey interviews demonstrate that respondents can misinterpret ordinary expressions in questions (like “bedroom” and “work for pay”)—that is, interpret them differently than the survey designers intend—much more often than one might think (Conrad and Schober, 2000), because the mapping or “fit” between their circumstances and the question concepts may not be straightforward (consider someone whose room originally designed as a den is being used as a bedroom, or whose freelance work included pay-in-kind). This is particularly a problem when interviews are strictly standardized; in more collaborative or “conversational” interviews, where interviewers and respondents work together to make sure respondents understand questions as intended (e.g., Schober and Conrad, 1997; Conrad and Schober, 2000), respondents generally interpret questions much more accurately. The best response accuracy, overall, seems to result not only when respondents request clarification if they believe they need it (“What do you mean by work for pay exactly?”), but when interviewers can also volunteer clarification when they believe respondents need it (Schober et al., 2004).

When designing a virtual interviewer for these kinds of surveys, a key consideration is, therefore, which features will best help respondents understand the questions as they are intended. Based on what is known about respondent comprehension in human-administered interviews, a virtual interviewer that can clarify question meaning when explicitly asked to do so and when it determines the respondent would better understand the question if its meaning were clarified—what we will call here a virtual interviewer with greater *dialog capability*—should, in principle, lead to more accurate comprehension. Whether this is actually the case with a virtual interviewer has not been demonstrated. Evidence from other automated implementations of survey interviews suggests that it could be the case, but it is not a foregone conclusion that it will be. For example, respondents' accuracy in a text-based web survey (Conrad et al., 2007) and in a (wizarded) spoken dialog survey system (Ehlen et al., 2007) improves when the system can provide clarification after a long period of inactivity or silence, but it does not improve in conditions where the only way to obtain clarification is to explicitly request it.

Whether high dialog capability interviewing systems with a facial representation will similarly promote comprehension is unclear. The addition of a face to the interface could make respondents even more reluctant to request clarification about ordinary words like “bedroom” and “job,” as they sometimes seem to be with human interviewers (Schober et al., 2004). Or, on the other hand, it could make them think the automated interviewer has greater agency and capabilities, and is thus better positioned to engage in clarification dialog. Because users' attributions about animated agents are likely to vary depending on the characteristics of the face—both static and dynamic (e.g., McDonnell et al., 2012; Piwek et al., 2014)—one might expect that survey response accuracy could be affected by how an animated virtual interviewer is visually implemented: survey respondents may evaluate the agent's competence and its likelihood of being able to provide useful clarification as greater when it behaves in a more human-like way. That is, they might assume that a more human-like face on a virtual interviewer means that the interviewer will comprehend requests for clarification better, and that the interviewer may better perceive the *respondent's* paralinguistic and facial displays of need for clarification (Schober et al., 2012).

### Hypotheses

In the study reported here, we test the following hypotheses about how a virtual survey interviewer's dialog capability and facial characteristics affect respondents' comprehension (as measured by the accuracy of their answers—our primary measure of task success). We also test how these factors affect respondents' social engagement with the interviewer, as measured by their behavioral displays as well as their subjective assessments of the interviewer. The facial characteristic that our hypotheses focus on is motion or *facial animation*: whether the face moves in a more or less human-like way, that is, with more or fewer channels of motion. We examine facial animation because this strikes us an attribute that is particularly likely to affect respondents' interpretation of a virtual interviewer's humanness; this is consistent with evidence in other task contexts that users interpret an embodied agent's intentions based more on audio and animation than on the render style of the character (McDonnell et al., 2012).

#### Hypotheses about comprehension

*Hypothesis 1*: *Dialog capability and comprehension*. A virtual interviewer with greater dialog capability will improve respondents' comprehension of survey questions, particularly when the fit between terms in the survey questions and the circumstances respondents are answering about is not straightforward.

This hypothesis will be supported to the extent that respondents treat a virtual interviewer with high dialog capability as better able than a low-dialog-capability virtual interviewer to interpret (1) their explicit requests for clarification and (2) indirect evidence of comprehension difficulty, both spoken and visual. If dialog capability affects comprehension in this way, its effect should be measurable both by response accuracy and by the number of requests for clarification. The basic mechanism is that more clarification should correct more misconceptions and resolve more ambiguities; the effect of dialog capability should be most evident when comprehension problems of this sort are most frequent, i.e., when the virtual interviewer asks questions about concepts that correspond in an ambiguous way to respondents' circumstances or whose definitions run counter to respondents' intuitions. We manipulate this experimentally in the study reported here.

The evidence to date that evaluates the effect of virtual survey interviewers on the quality of responses does not provide evidence about whether dialog capability works the same way or to the same extent with human and virtual interviewers. For example, while the Lind et al. (2013) study concerned survey interviews, the authors did not design the virtual interviewers to provide clarification; moreover the interaction was not entirely spoken: the interviewing agents asked questions orally but respondents answered by clicking or typing. If clarification does not work the same way—if respondents don't solicit or interpret clarification in the same way—with virtual interviewers in a spoken dialog interview as they do with human interviewers, the hypothesis will not be supported. This could occur if, for example, respondents do not treat the virtual interviewer as conversationally competent—which might be affected by the interviewer's facial animation.

*Hypothesis 2*: *Facial animation and comprehension*. A virtual interviewer with more facial animation will improve respondents' comprehension of survey questions.

This hypothesis will be supported if survey respondents attend better or try harder at the survey response task when an interviewer seems more human-like, which can result from a virtual agent's increased motion (Hyde et al., 2013; Piwek et al., 2014). The evidence is that perceiving another person's facial motion *can* improve at least some kinds of task success. For perceptual tasks, for example, people tend to be better at detecting a speaker's identity when presented with a moving than a static face (see Xiao et al., 2014, for a review), and they can comprehend speech even in noisy conditions better with facial (especially mouth) motion cues than without (Alexanderson and Beskow, 2014). In avatar-mediated communication, participants are better able to detect truth and deception when an avatar has realistic eye motion (Steptoe et al., 2010).

On the other hand, in a survey interview setting where the measure was disclosure of sensitive information rather than comprehension accuracy, Lind et al. (2013) found less disclosure to a high-motion virtual interviewer than to a low-motion interviewer for some survey questions, and no difference in disclosure for others. To the extent that these disclosure findings are relevant to comprehension and response accuracy for non-sensitive survey questions, increased facial motion in a virtual interviewer may not improve survey task performance, and this hypothesis will not be supported.

*Hypothesis 3: Interactive effects of facial animation and dialog capability on comprehension*. A virtual interviewer with more facial animation may improve respondents' comprehension of survey questions particularly when the interviewer has greater dialog capability. To put it another way, a virtual interviewer's dialog capability may improve comprehension particularly when the interviewer's facial animation is consistent with greater dialog competence.

If a virtual interviewer's greater facial animation suggests that it has greater dialog competence, respondents may be particularly more likely to seek clarification (explicitly request it) or to provide indirect evidence of their need for clarification (paralinguistic or facial), and thereby comprehend and answer more accurately, than if an interviewer has less facial animation. If so, this would predict an interaction: greater clarification-seeking or evidence of need for clarification, and thus improved response accuracy, with a high-animation agent in a high-dialog-capability condition.

On the other hand, greater facial animation could lead to unrealistic expectations that the agent's dialog competence is fully human, which could subsequently conflict with the agent's actual abilities; in this case, greater facial animation could, paradoxically, lead to poorer comprehension if the respondent relies solely on the interviewer to diagnose need for clarification. One could also imagine other interactive effects: an interviewer with low facial animation might lead users to *under*estimate the dialog capability of high-dialog-capability agents, and thus request clarification or produce indirect evidence of need for clarification less often than would be optimal.

Although hypotheses about interactive effects of a virtual interviewer's dialog capability and facial animation have not been tested before, the plausibility of such effects is strengthened by the finding that survey respondents in face-to-face interviews produce more paralinguistic displays of need for clarification (speech disfluencies) and avert their gaze more often for unreliable answers in high-dialog-capability (conversational) than low-dialog-capability (strictly standardized) interviews (Schober et al., 2012). Of course, human interviewers have high facial animation in the sense we are exploring here, unless their facial mobility is impaired from neurological illness or cosmetic interventions, and yet when they conduct standardized interviews they are required to restrict their ordinary dialog capability; so a mismatch between facial animation and dialog capability is not unusual in human-administered survey interviews. On the other hand, if comprehension in surveys depends mostly on the conceptual content conveyed by dialog, the interviewer's facial animation will not interact with dialog capability in affecting respondents' comprehension.

#### Hypotheses about engagement

Independent of comprehension or clarification-seeking behavior, a virtual interviewer's dialog capability and facial animation could have independent or interactive effects on survey respondents' engagement with the interview, as evidenced by their social behaviors during the interaction (e.g., time spent looking at the virtual interviewer, nods and verbal acknowledgments, and smiles) and by how they experience the interview subjectively.

Respondents' engagement in survey interviews—their involvement, attentiveness, and conscientiousness—is critical for obtaining accurate data. But respondents can be less engaged in the interview task than would be desirable, perhaps because most do not ask to be interviewed (the researchers invite them via an interviewer). In conventional survey modes, evidence of respondents' lack of engagement can be seen in their terminating an interview before it is completed (see Peytchev, 2009 for a discussion of breakoffs in online questionnaires) and in their least-effort “satisficing” as they answer questions, for example selecting the same response option again and again in a battery of questions (e.g., Chang and Krosnick, 2010). Our focus here is on respondents' behavioral displays of engagement during the course of a virtual interview—their gaze, their spoken and visual acknowledgments, and their smiles—and their reported post-interview assessments of their interview experience.

With this focus, we test the following hypotheses:

*Hypothesis 4: Dialog capability and engagement*. A virtual interviewer whose interaction is more like everyday conversation—who can clarify the questions—will engage respondents more than a virtual interviewer with low dialog capability.

One might expect that when survey respondents interact with a virtual interviewer with more human-like capabilities they will behave more as they do in ordinary conversation: they will look at their interlocutor more, acknowledge their understanding more (nod, produce backchannels like “okay”), display social cues (smile), and rate the interaction as more positive. To our knowledge this has not been examined directly, but accounts of frustration experienced by respondents whose standardized interviewers are prevented from providing clarification (e.g., Suchman and Jordan, 1990) are consistent with this hypothesis.

*Hypothesis 5: Facial animation and engagement*. A virtual interviewer whose facial movement is more human-like will engage respondents more than a virtual interviewer with low facial animation.

From other domains of interaction with virtual agents, the evidence is that people judge agents with more (bodily) motion as more acceptable and human (Piwek et al., 2014), and that realistic characters that move more are judged more positively (Hyde et al., 2013). The benefits of more human-like behavior may well extend to the survey context: Conrad et al. (2013) demonstrated that people invited to participate in (human-administered) telephone survey interviews were more likely to agree to participate when the interviewers spoke less robotically (with more disfluencies) during the invitation interaction. And Foucault Welles and Miller (2013) demonstrated that respondents in face-to-face (human-administered) survey interviews reported feeling greater rapport (which is presumably related to their feelings of engagement) when *interviewers* nodded and smiled more, and when they gazed at respondents' faces less.

*Hypothesis 6: Interactive effects of facial animation and dialog capability on engagement*. A virtual interviewer with more facial animation may increase respondents' engagement particularly when the interviewer has greater dialog capability.

Any effects of dialog capability and facial animation on respondents' display of social cues or assessment of the interviewer could be independent, or they could interact. The same range of possible interaction effects exists for measures of engagement as for comprehension. The combination of low dialog capability and low facial animation could lead to particularly unengaging or alienating interaction. High facial animation could lead to unrealistic expectations about an interviewer's dialog capability, which when thwarted could lead respondents to be *less* engaged with the interviewer. Low facial animation could lead to underestimation of a high dialog capability interviewer's competence, which could lead respondents to attend less fully to or disengage with the interviewer.

## Materials and methods

Our strategy in this study was to bring participants to our laboratory to respond to 12 questions about housing, work and purchases taken from US government surveys, which they answered on the basis of scenarios describing fictional circumstances. This allowed us to directly assess the accuracy of their responses—which also measures the extent to which their comprehension of the terms in the survey questions fits what the official definitions of those terms would require. Participants (respondents) were randomly assigned to be interviewed by a (Wizard-of-Oz) interviewing agent with either high or low facial animation (many channels/points of motion vs. few) and high or low dialog capability (conducting interviews in either a collaborative or strictly standardized style). For each respondent, half the fictional scenarios were designed to map onto the survey questions in a straightforward way and half in a complicated way. Thus, the experimental design was 2 × 2 × 2.

Although having respondents answer about fictional scenarios as opposed to about their own lives reduces ecological validity, it has the advantage of allowing direct assessment of accuracy of comprehension during the interviews. In other studies with human interviewers we have used post-interview self-administered questionnaires (Suessbrick et al., 2000; Schober et al., 2012) and human-administered re-interviews (Conrad and Schober, 2000; Suessbrick et al., 2000) as alternate (less direct) methods for assessing comprehension and survey response accuracy, under the logic that response change when respondents are provided with a standard definition of a survey term is likely to reflect the correction of a misinterpretation in the original interview; the findings in those studies are highly consistent with the findings produced when responses are based on fictional scenarios, and so in the current study we use fictional scenarios. The questions and scenarios in the current study are the same as those used in previous laboratory studies of telephone interviews (Schober and Conrad, 1997; Schober et al., 2004) and of online text- and speech-based interviewing systems (Conrad et al., 2007; Ehlen et al., 2007). Although the participant sample and time frame for this experiment make a comparison with those studies not entirely parallel, they provide relevant context for evaluating respondents' performance with a virtual interviewer in the current study.

### Experiment materials

#### Survey questions

The 12 survey questions were adapted to apply to the fictional scenarios that respondents would be answering about, rather than about the respondent's own circumstances: four questions about employment from the US Current Population Survey (e.g., “Last week, did Chris do any work for pay?” filling in the name of the fictional character Chris in the question “Last week, did you do any work for pay?”), four questions about purchases from the Current Point of Purchase Survey (e.g., “Has Kelly purchased or had expenses for household furniture?”), and four questions about housing from the Consumer Price Index Housing Survey (e.g., “How many bedrooms are there in this house?”). Each question had a corresponding official definition for its key concepts developed by the sponsoring agency. For example, for the question “Has Kelly purchased or had expenses for household furniture,” the official definition of household furniture is this:

Tables, chairs, footstools, sofas, china cabinets, utility carts, bars, room dividers, bookcases, desks, beds, mattresses, box springs, chests of drawers, night tables, wardrobes, and unfinished furniture. Do not include TV, radio, and other sound equipment, lamps and lighting fixtures, outdoor furniture, infants' furniture, or appliances (US Bureau of the Census and US Bureau of Labor Statistics, 1993).

(Supplementary Table 1 includes all questions and the official definitions relevant to each question).

The questions were ordered for the experiment to correspond with the order in which they appeared in the survey from which they were drawn, and counterbalanced across domains for different respondents to make sure that any effects observed in the experiment could not be attributed to the order in which the virtual interviewer asked about the different domains. So one respondent would answer purchase questions followed by housing questions followed by employment questions, another would answer housing questions followed by employment questions followed by purchase questions, etc.

#### Respondent scenarios

Fictional scenarios on the basis of which respondents were to answer the questions were assembled into paper packets, with one page per scenario. In actual surveys respondents most often answer based on their recall and self-assessment; using scenarios is more similar to situations when respondents answer by consulting their personal records, and, more importantly, allows us to isolate and focus on comprehension—there is no autobiographical recall involved when respondents answer based on scenarios. For factual questions about respondents' behaviors or circumstances, the outcome of each exchange—an answer to a survey question—is either accurate or not (e.g., the respondent either has or has not done any work for pay in the last week). In principle this could be independently assessed if researchers were to have independent evidence about the respondent's circumstances (e.g., trustworthy records from the respondent's place of employment), but of course, in many cases (e.g., for many personal behaviors and for respondents' opinions) there is no independently verifiable evidence about the accuracy of responses.

The scenarios, which were not seen by the interviewing Wizard during the interview, consisted of work descriptions, purchase receipts, and floor plans. Two alternate scenarios were created for each question, one describing situations that mapped onto questions and the corresponding official definitions in a straightforward way (“straightforward mappings”) and one describing situations that mapped onto questions and official definitions in a complicated way (“complicated mappings”)—for which respondents might well need clarification in order to answer the question in a way that fit the definition. For example, for the question about household furniture, the straightforward scenario was a receipt for the purchase of an end table. The complicated scenario was a receipt for the purchase of a floor lamp. The official definition—which was not part of the materials given to the respondents, but could only be presented orally by a high-dialog-capability virtual interviewer—clarified that for the purposes of this survey, a floor lamp is not to be counted as a household furniture purchase, and thus the answer to this question should be “no.” (The answer for the straightforward scenario should be “yes,” as an end table counts as a furniture purchase).

The selection of these scenarios thus allowed direct evaluation of whether the respondent had comprehended the question in a way that fit the official definitions. A respondent who answers “yes” to the household furniture question with a floor lamp receipt, or “no” with an end table receipt, is not interpreting the question as the survey designers intended; these responses can be classified as incorrect.

Scenario packets were assembled for each respondent that included half (6) straightforward and half (6) complicated scenarios, with two straightforward and two complicated scenarios per domain (employment, purchases, housing). The orderings of mappings were counterbalanced across respondents, such that the particular combination of straightforward and complicated mappings for one respondent was the complement of the combination for another. Across all respondents, both straightforward and complicated scenarios were presented equally often and in different orders, both so that the interviewing Wizard could not anticipate which scenario a particular respondent was encountering and so that any effects observed in the experiment could not be attributed to a particular sequence of mappings.

#### Additional interviewer utterances

In addition to the survey questions and the full definitions of relevant terms in the questions, all other allowable interviewer utterances in low and high dialog capability interviews were scripted. These included several introductions of the interview (e.g., “Hello, my name is Derek and today I will be asking you a few questions about housing, jobs and purchases.”), pre-interview practice material, neutral probes (e.g., “Is that a yes or a no?”), partial definitions (just the text that resolves the ambiguity in the corresponding complicated scenario), clarification offers (“It sounds like you're having some trouble. Can I give you a definition that might help?”), utterances to manage the dialog (e.g., “Yes,” “No,” “Please wait one moment”), and utterances to run the experimental session (“Please turn to the next page of your packet”; “I am going to ask the research assistant to help you. Just a minute please”). Supplementary Table 2 lists the full set of additional scripted utterances.

### Developing the virtual interviewers

**Figure 1:**
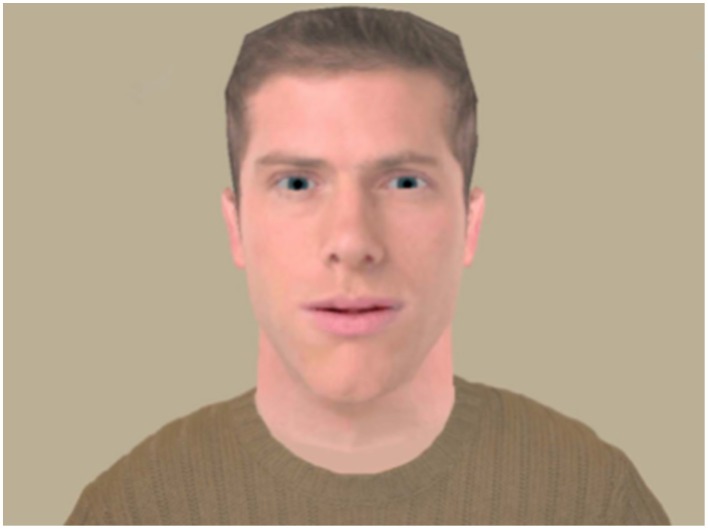
**The Derek model that formed the basis of the four virtual interviewers**.

The virtual interviewers for the four experimental conditions were created using famous3D's ProFACE video software (version 2.5) to make variants of a single 3D model of a head. We first video- and audio-recorded a human interviewer (a male graduate student in survey methodology who spoke American English) administering all survey questions, prompts, clarifications, and additional interviewer utterances, with 21 green and blue dots affixed to his face to capture 21 different motion channels (forehead, outer and inner brows, furrow, upper eyelids, region below the eyes, cheeks, right and left sides of nose, right and left lower lips, chin, etc.). With the ProFace software we captured his facial motion and mapped it to a face template, which could then be projected onto one of ProFace's existing models (Derek, in our case; see Figure 1) either using all motion channels (for the high facial animation conditions) or a subset (for the low facial animation conditions). All audio files used in the low dialog capability conditions were also used in the high dialog capability conditions; there were, of course, extra speech files (and accompanying video) for high dialog capability conditions (e.g., offers of clarification).

Note that because all four virtual interviewers were based on the same head model, the interviewer's base level of visual realism or naturalism, which can affect how users judge and respond to virtual agents in other task contexts (e.g., Baylor and Kim, 2004; MacDorman and Ishiguro, 2006; Gong, 2008; MacDorman et al., 2009), was the same across all four conditions. In a job interview training task, Baur et al. (2013) found that interviewees criticized their interviewer as not looking realistic enough; our interviewer has a level of realism that reflects the photographic origins of the model, and is more realistic than the more cartoon-like survey interviewer in Lind et al. (2013), but there is simply not enough evidence in survey tasks about the optimal levels of realism for a virtual survey interviewer.

Also note that because the interviewers differed behaviorally on more than one feature, any effects on respondents must be attributed to bundles of behavioral features rather than individual features.

#### Facial animation

Table 1 summarizes the major features of motion in the high and low facial animation interviewers. For the low facial animation conditions, seven motion channels were projected onto the Derek model: chin, left and right lower lips, left and right corners of mouth, and left and right peaks of lip. The low animation interviewer head and face do not move, the eyes do not blink, and the mouth does not change shape as the interviewer speaks—it just opens and closes.

**Table 1:** **Facial animation features of virtual interviewers**.

	**Low facial animation**	**High facial animation**
Head moves	No	Yes, even when “listening”
Face moves	Only mouth	Yes
Eyes move	No	Yes
Eyes blink	No	Yes
Mouth movement	Only opens and closes during speech, but does not change shape	Mouth forms appropriate shapes for sounds being produced

For high facial animation conditions, in addition to the 21 channels of captured motion the interviewer's head and face move (applying ProFace's jitter function) at all times (even while waiting for responses, to give the appearance of listening), and his eyes blink. The interviewer's mouth forms appropriate shapes for the sounds he is producing; to improve the correspondence between the interviewer's mouth movements and speech, additional keyframes were added by hand beyond the captured motion at a fine level of granularity, with particular combinations of motions for different consonants and vowel sounds in the recordings, based on the judgments of an animator. Finally, stationary shoulders were added to make the head movements look more realistic.

See Supplemental Data for video examples of low and high facial animation introductions to the interview (Videos 1, 2) and for low and high facial animation variants of Purchases Question 3 (Videos 3, 4).

#### Dialog capability

Table 2 summarizes the major features of dialog capability in the high and low dialog capability interviewers. These were implemented by an experimenter behind the scenes (the Wizard) following protocols for which interviewer files (questions, neutral probes, definitions, etc.) were to be played to respondents in which sequence and in response to which respondent behaviors (see Wizard protocols below). In all cases the virtual interviewers presented the same questions, and (from the respondents' perspective) they could comprehend and register spoken answers.

**Table 2:** **Dialog capability features of virtual interviewers**.

	**Low dialog capability**	**High dialog capability**
Reads question as worded	Yes	Yes
Understands spoken answers	Yes	Yes
Repeats question if asked	Yes	Yes
Understands explicit requests for clarification	Yes	Yes
Provides clarification when explicitly requested	No: presents neutral probe (e.g., “Whatever it means to you”; “Let me repeat the question”)	Yes: reads definition
Offers clarification when it seems needed (based on respondent's verbal and visual behavior)	No	Yes

The low dialog capability protocol was to administer a strictly standardized interview, as implemented in previous studies in this line of research (e.g., Schober and Conrad, 1997; Schober et al., 2004). The virtual interviewer presented the questions exactly as worded and could repeat questions if asked, but if a respondent explicitly requested clarification the interviewer would only provide a neutral probe (of the Wizard's choosing, just as in human-administered standardized interviews; see Video 5 in Supplementary Materials for an example).

The high dialog capability protocol was to administer “conversational” interviews, again as in Schober and Conrad (1997). After reading the question exactly as worded, the (wizarded) interviewer (a male graduate student) did whatever he thought was needed to make sure that the respondent had interpreted the question as intended—to “ground” the meaning of terms in survey questions, to use Clark and colleagues' term (e.g., Clark and Wilkes-Gibbs, 1986; Clark and Schaefer, 1987; Clark, 1996). In other words, the interviewer's task was to make sure that the respondent's interpretation fit the official definition. This included not only providing the full official definition if the respondent explicitly requested it but also offering clarification if the interviewer (Wizard) got the sense that the respondent might need it (see Video 6 in Supplementary Materials for an example). Given the nature of the video files and wizarding protocols, this implementation of conversational interviewing is not as fully flexible as human interviewers can provide, because our virtual interviewers could not provide fully tailored partial definitions or improvise unscripted dialog, but it is on the most flexible end of the continuum (see Schober et al., 2004).

#### Pre-study: verifying distinctiveness of virtual interviewers

In order to increase our confidence that we had successfully manipulated what we hoped to in creating the virtual interviewer videos, we collected ratings of all 130 video clips in the experiment, both low and high facial animation versions. The clips included all questions, probes, definitions, and introductions to be used by both the low and high dialog capability virtual interviewers. Thirteen raters (11 female, two male; mean age 28.8, ranging from 24 to 34; all with bachelors' degrees, six graduate students in survey methodology) each rated 65 high- and low-animation video clips in one of two group viewing sessions. For each clip, each rater judged the virtual interviewer on a ten point scale for warmth (“How warm was Derek, with 0 being Not At All Warm and 10 being Very Warm?”), naturalness (“How natural was Derek, with 0 being Not At All Natural and 10 being Very Natural?”), and similarity to an actual interviewer (“To what degree did Derek seem like an actual interviewer, with 0 being Not At All Like An Interviewer and 10 being Very Much Like An Interviewer?”).

The ratings confirmed that the high facial animation virtual interviewers were, in the aggregate, perceived to be reliably warmer [4.58 vs. 2.78 on the 10-point scale, *F*_(1, 12)_ = 28.56, *p* < 0.001, η^2^ = 0.704], more natural [5.23 vs. 2.95 on the 10-point scale, *F*_(1, 12)_ = 36.24, *p* < 0.001, η^2^ = 0.751], and more like a human interviewer [6.24 vs. 4.36 on the 10-point scale, *F*_(1, 12)_ = 21.35, *p* = 0.001, η^2^ = 0.640] than the low realism versions. The same pattern was observed for most individual clips, though not all. Although none of the ratings reached the top of the 10 point scale, these strongly reliable differences suggested to us that these implementations of virtual interviewers would be suitable for the experiment.

### Wizarding protocols

The virtual interviewers were controlled by mapping each video file to a key on the computer keyboard using ArKaos VJ software. This allowed the Wizard to present the next relevant file to the respondent by pressing a key, according to the relevant protocol for high or low dialog capability interviewing (see Table 3 for the Wizard's decision rules). Using the VJ software allowed seamless presentation of the video clips, so that the virtual interviewer appeared to the respondent to be acting on its own. The Wizard sat in a control room with a one-way mirror and live video feed view of the respondent. The control computers were set up so that the Wizard could view the respondent from a frontal overhead position and could also see the video file of the virtual interviewer as it was playing for the respondent.

**Table 3:** **Wizard's decision rules**.

**Low dialog capability**	**High dialog capability**
Give respondent 3 min to familiarize him/herself with packet, and ignore respondent if he/she says he/she is ready	Give respondent 3 min to familiarize him/herself with packet, but begin interview if respondent says he/she is ready
Wait 10 s between transition and question clip, despite respondent behavior	Wait for respondent to look at virtual interviewer before presenting next question clip
Do not modify presentation of clips based on respondent's gaze or attention	Stop presenting a clip if respondent stops looking at virtual interviewer
Send research assistant to help respondent if in trouble	Use virtual interviewer to assist respondent if in trouble. If not successful send research assistant
If respondent seems hesitant or confused, do nothing	If respondent seems hesitant or confused, then offer help
If respondent asks for help, then present neutral probe	If respondent asks for help not related to scenario, then present neutral probe
	If respondent asks for help pertaining to scenario, then present entire definition
	If respondent asks for help with specific mention of key concept, then present partial definition
If respondent interrupts virtual interviewer, then finish presenting clip. Wait for respondent to repeat him/herself	If respondent interrupts virtual interviewer, then present waiting clip and address respondent's concern immediately

The use of a Wizard allowed us to implement the high and low dialog capability virtual interviewers without programming a full survey dialog system with speech recognition and dialog management, which was beyond the scope of the current study [In other projects we have implemented a standardized survey spoken dialog system for mobile devices (Johnston et al., 2013) and experimented with an automated telephone system that implements conversational interviewing, including modeling respondents' paralinguistic displays of need for clarification (Ehlen et al., 2007)]. Because the same Wizard manipulated the virtual interviewers in this study across all conditions, his detection of and judgments of the meaning of respondents' facial and bodily displays and verbal behavior were likely to be consistent in the different conditions. This means that across the high and low facial animation conditions, the timing of turn transitions (the point at which speakers and listeners trade roles in conversation), which has been shown to affect perceptions of (in particular rapport with) virtual humans (Huang et al., 2011), were deployed based on the same human Wizard judgments, appropriately for either the high or low dialog conditions. Thus, although by necessity the Wizard needed to be informed about respondents' experimental conditions (so that he could deploy the appropriate video files), the particular linguistic and interactive intuitions that the Wizard brought to the experiment did not differ across the conditions.

### Post-interview measures

After completing the interview, respondents filled out an online questionnaire in which they reported their subjective experience interacting with the virtual interviewer on a number of dimensions (e.g., “How much did you enjoy interacting with Derek?”, “Would you say that Derek acted more like a computer or a person?”, “How often did Derek seem to act on his own?”). They also provided information about their technological experience (“How often, on average, do you use a computer?”) and their demographic and linguistic background (e.g., “Is English your native language?”). The full questionnaire is presented in Supplementary Table 3.

### Participants

Seventy-five participants (respondents) were recruited from the local site of the Craig's List online forum (https://annarbor.craigslist.org/) (*n* = 51) and through word of mouth (*n* = 21); for three respondents we do not have records about how they heard about the study.

Respondents, who were paid $35 for participating, were each randomly assigned to an experimental condition, except for two who were recruited specifically to replace two respondents (one in each high-dialog-capability condition) who expressed suspicion that the virtual interviewer was wizarded (the replaced and replacement respondents were all recruited through Craig's List). This led to a final data set with 18 respondents in three of the four conditions and 19 in the high-dialog-capability-high-facial animation condition.

In the final data set, the composition of the four groups did not differ reliably in age (*F* < 1), nor in recruitment source (*p*-values for all **X**^2^>0.15.) The respondents ranged in age from 18 to 67 years (mean = 36.8); 38 were female and 35 were male. 56.2% of respondents reported being White, 20.5% Black, 16.4% Asian or Pacific Islander, and 5.5% reported being members of other groups. 37.4% of respondents reported their highest level of education as less than a bachelor's degree, 42.5% as a bachelor's degree, and 19.2% as a graduate or professional degree. As a group they were highly computer literate, with 84.9% reporting using a computer 5–7 days per week. 89% reported that English was their native language.

All procedures that respondents followed, and all materials that were presented to them, were reviewed and approved by the University of Michigan IRB-HSBS (Institutional Review Board—Health Sciences and Behavioral Sciences).

### Procedure

Each respondent was escorted to a first room where he or she signed consent forms and was handed the packet of experimental scenarios on the basis of which he or she would be answering survey questions. A research assistant instructed respondents using the following script:

In this study, you will be asked 12 questions about fictional purchases, housing, and jobs. This interview is not like typical interviews. We will not be asking you about your own experiences but about the information contained in scenarios in this packet, so we can assess the accuracy of your responses. On each page there is one scenario, which corresponds to one question. You should answer each question based only on information in the corresponding scenario. Each scenario is independent of each other, so you should *not* use information from the previous page to answer a subsequent question. Some of the scenarios are dated; consider the date in the packet to be current, rather than responding based on today's date. You will receive additional information about this procedure once the interview begins. Let's enter the room now to start the interview.

Respondents were then led to a second room, which contained two mounted cameras, a chair, a table, a computer, a monitor displaying the virtual interviewer, a microphone on the table, and (in the high-dialog-capability conditions) a non-functioning web camera trained on the respondent to increase the plausibility that the virtual interviewer could sense the respondent. The room was free of other distractions. If a respondent asked about any of the equipment, the research assistant answered by saying, “I will be happy to answer your questions after the interview.” The research assistant then pointed at the monitor with the virtual interviewer and gave the following instructions:

You are going to be interviewed by Derek. Derek will speak to you, and you should respond aloud. Please look at Derek when he's speaking to you. Okay?

When I leave the room, Derek will introduce himself and give you the opportunity to familiarize yourself with the scenario. Please use all the available time to fully acquaint yourself with the entire packet. You may also want to review each scenario before answering its respective question.

This is a new way to conduct interviews and, therefore, might be a little rough around the edges. Please bear with us if there are any problems. Let me know if you experience any difficulty with the equipment. I am leaving now, but please feel free to knock on the door if you need my help. The interview will begin as soon as I leave the room. Any questions?

In the high-dialog-capability conditions, the research assistant presented the following additional instructions:

Please look at Derek when you are ready for the next question. Derek can hear and see you.

Sometimes, survey questions use ordinary words in unexpected ways. To be sure you understand the question, you may need to ask Derek to clarify particular words so please ask for clarification if you are *at all* unsure about what they mean. In fact, you may need to get clarification from Derek in order to answer accurately. Unlike what happens in some survey interviews, Derek *is* able to help you when you indicate you need help. So you should be sure to ask Derek for clarification if you are at all unsure about what a word means.

This description of the respondent's role in conversational interviews parallels the additional instructions in Schober and Conrad (1997).

The research assistant then left the room and the interview proceeded, starting with a first training question and scenario to familiarize the respondent with the task. The research assistant, who monitored the video and audio of the interview along with the Wizard, was available to enter the room if there were technical difficulties or if the respondent gave evidence of not having understood the instructions (e.g., about turning the page in their scenario packet for each next survey question).

After the interview, the research assistant escorted respondents to another lab room, where they filled out the on-line post-experiment questionnaire. Finally, they were asked whether they felt they were indeed interacting with a computer (to give them the opportunity to voice any suspicions that the virtual interviewer was wizarded), debriefed about the actual Wizard-of-Oz experiment setup, and paid for their participation.

The reported analyses are based on the 73 respondents who gave no evidence in the experiment debriefing of suspecting that the virtual interviewer was wizarded. From transcripts of the interviews, we know that no participant ever expressed any suspicion or asked any questions about how the virtual interviewer worked during the interview.

## Results

### Comprehension

To test our Hypotheses 1–3 about comprehension, we first focus on response accuracy and then on respondents' and virtual interviewers' clarification behaviors. We adopt conventional thresholds for alpha, with levels of *p* < 0.05 as statistically significant (reliable) and 0.05 < *p* < 0.10 as marginal.

#### Response accuracy

Respondents' comprehension was measured by observing, for each response, whether it matched what the official definition of the survey term would require.

**Figure 2:**
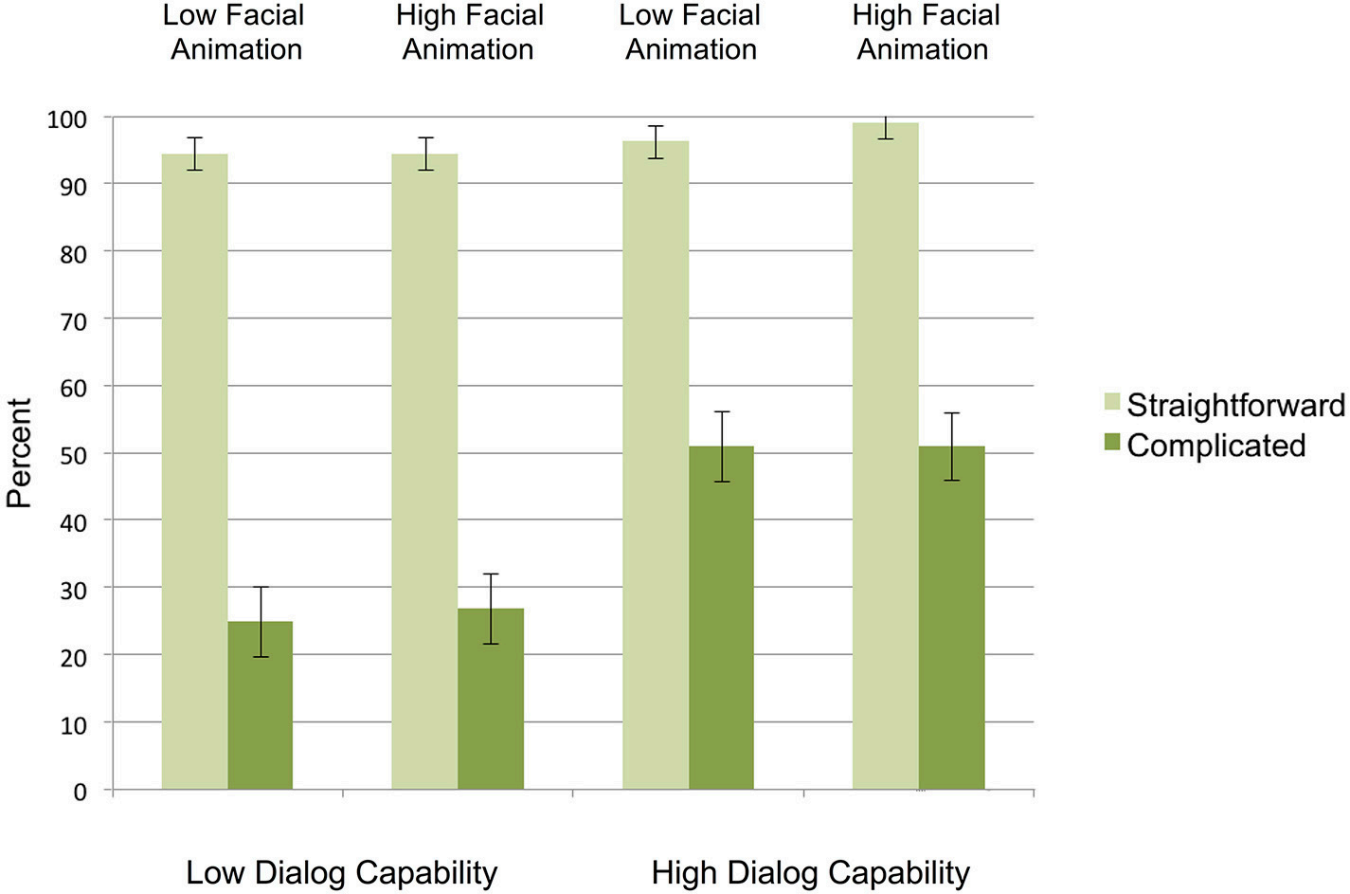
**Response accuracy (percentage of survey responses that matched what the official definition would require) for straightforward and complicated scenarios (error bars represent SE's)**.

As Figure 2 shows, Hypothesis 1 was supported: virtual interviewers with high dialog capability led to significantly greater response accuracy (74.3%) than virtual interviewers with low dialog capability (60.2%), *F*_(1, 69)_ = 21.69, *p* < 0.001, η^2^ = 239. This was entirely driven by the effect of dialog capability on response accuracy for complicated mapping scenarios (50.9% for high dialog capability and 25.9% for low dialog capability interviewers); in contrast, for straightforward mappings there was no effect of interviewer dialog capability on response accuracy (response accuracy was uniformly high in all conditions), as demonstrated by the interaction of mapping by dialog capability *F*_(1, 69)_ = 15.38, *p* < 0.001, η^2^ = 0.182.

Figure 2 also shows that, contrary to Hypothesis 2, there was no evidence that the virtual interviewer's facial animation affected response accuracy, *F*_(1, 69)_ = 0.15, *p* = 0.70, η^2^ = 0.002. To further investigate whether there really was no effect of facial animation on response accuracy, we computed a Bayes_10_ factor (using the JASP, 2015 package) comparing the fit of the data under the null hypothesis (no effect of facial animation) and the alternative (see Jarosz and Wiley, 2014 for an account of the underlying logic). An estimated Bayes_10_ factor (alternative/null) of 0.193 suggested that the data were 5.18:1 in favor of the null hypothesis, that is, 5.18 times more likely to occur under a model without including an effect of facial animation, rather than a model with it (in comparison, an estimated Bayes factor [alternative/null] for dialog capability is 2.092 in favor of the alternative hypothesis).

Contrary to Hypothesis 3, and further supporting the interpretation that the virtual interviewer's dialog capability was entirely responsible for response accuracy, is the finding that the interaction between response accuracy and facial animation was not significant, *F*_(1, 69)_ = 0.006, *p* = 0.94, η^2^ = 0.000; the Bayes_10_ factor for the interaction between dialog capability and facial animation is 0.386, suggesting that the data are 2.59:1 in favor of the null hypothesis.

#### Clarification behaviors

So that we could examine direct and indirect requests for clarification and their relationship with respondents' comprehension, complete transcripts of the survey question-answer sequences in each interview were created and coded. A coding scheme for all interviewer and respondent moves (see Supplementary Table 4) was adapted from our previous studies with human interviewers (Schober et al., 2004) that included codes for the behaviors we expected to differ between high- and low-dialog-capability interviews (e.g., offering clarification, providing definitions, providing neutral probes). In order to verify reliability of the coding, the majority of the question-answer sequences (86.6%) were coded again by a different coder; agreement between these two sets of codes was measured with Cohen's kappa, which at 0.988 was “almost perfect” by Everitt and Haye's (1992, p. 50) characterization.

Consistent with Hypothesis 1, respondents only ever requested or received clarification in the high dialog capability conditions, and not at all in the low dialog capability conditions. This makes sense because of course any requests with a low-dialog-capability virtual interviewer would be met with a neutral probe (e.g., “Let me repeat the question” or “whatever it means to you”) rather than substantive clarification (e.g., “In this survey we do not include floor lamps as furniture”).

Also consistent with Hypothesis 1 (see Table 4), respondents with the high-dialog-capability virtual interviewers explicitly requested clarification more often—nearly twice as often—for complicated scenarios than for straightforward scenarios, and they correspondingly received clarification more than twice as often for complicated scenarios. The virtual interviewer also was more likely to comment on the respondent's need for clarification for complicated scenarios. Compared to explicitly requesting clarification, respondents indirectly indicated that they were having comprehension trouble (e.g., “I don't know whether to count that or not”) far less frequently, and they did not do this at different rates for different scenario types.

**Table 4:** **Percentage of question-answer sequences in which clarification and related speech occurred (SE's in parentheses)**.

	**Scenario mapping**		**Effect**	**Facial animation**		**Effect**
	**Straight forward**	**Complicated**		**Low**	**High**	
Respondent explicit requests for clarification (“What do you mean by ‘furniture’?”)	**18.1 (3.7)**	**35.2 (5.2)**	***F*_(1, 35)_ = 20.74,** ***p* < 0.001,** **η^2^ = 0.372**	29.2 (5.9)	24.1 (5.8)	*F*_(1, 35)_ = 0.37, *p* = 0.55, η^2^ = 0.011
Respondent implicit requests for clarification (“I don't know whether to count that or not”)	6.3 (1.8)	4.4 (1.8)	*F*_(1, 35)_ = 0.88, *p* = 0.354, η^2^ = 0.025	4.6 (2.1)	6.2 (2.1)	*F*_(1, 35)_ = 0.27, *p* = 0.605, η^2^ = 0.008
Virtual interviewer comments on respondent's confusion (“It sounds like you're having some trouble.”)	**3.6 (1.2)**	**8.9 (1.7)**	***F*_(1, 35)_ = 8.08,** ***p* = 0.007,** **η^2^ = 0.188,**	**4.2 (1.6)**	**8.4 (1.6)**	***F*_(1, 35)_ = 3.42,** ***p* = 0.073** **η^2^ = 0.089**
Virtual interviewer offers clarification (“Can I help you?”)	25.8 (3.4)	25.2 (3.2)	*F*_(1, 35)_ = 0.022, *p* = 0.882, η^2^ = 0.001	**31.5 (3.8)**	**19.6 (3.7)**	***F*_(1, 35)_ = 4.98,** ***p* = 0.032,** **η^2^ = 0.124**
Respondent rejects offer	5.3 (1.6)	3.2 (1.1)	*F*_(1, 35)_ = 1.36, *p* = 0.251, η^2^ = 0.037,	5.1 (3.4)	3.4 (1.4)	*F*_(1, 35)_ = 0.67, *p* = 0.42, η^2^ = 0.019
Virtual interviewer presents definition	**16.3 (3.5)**	**36.6 (4.5)**	***F*_(1, 35)_ = 26.55,** ***p* < 0.001,** **η^2^ = 0.431**	29.6 (5.1)	23.3 (5.0)	*F*_(1, 35)_ = 0.80, *p* = 0.38, η^2^ = 0.022

*Statistically reliable and marginal differences are in bold face*.

Contrary to Hypothesis 2 (see Table 4), there was no evidence that respondents in the high dialog capability conditions explicitly requested clarification any more often when the virtual interviewer had high than low facial animation, nor did they reject clarification or receive definitions any more often.

Even though there was no evidence that the virtual interviewer's facial animation affected respondents' requests for clarification, respondents with high animation virtual interviewers did have different clarification dialog experiences in a few other ways. Respondents with the high animation virtual interviewer were marginally more likely to be presented with a comment about their confusion (“It sounds like you're having some trouble”) than respondents with the low animation virtual interviewer. This is potentially consistent with Hypothesis 2, to the extent that respondents' non-verbal or paralinguistic evidence of confusion (beyond explicit or implicit verbal requests for clarification) differed enough between high and low animation virtual interviewers so as to affect the Wizard's presentation of such comments. On the other hand, Hypothesis 2 seems clearly contradicted by the less interpretable finding that respondents with a high facial animation virtual interviewer were reliably *less* likely to be offered unsolicited clarification. This would make sense if we saw other evidence that respondents requested clarification or provided evidence of confusion more with the low facial animation interviewer, but that is not what we observe. In any case, although we see little evidence for Hypothesis 2, the fact that clarification dialog can proceed differently when the interviewer has high or low facial animation suggests that the impact of facial animation on clarification dialog deserves further exploration.

Analyses of potential interactive effects of the interviewer's dialog capability and facial animation on respondents' requests for clarification and receiving clarification are not significant. Consistent with the response accuracy evidence, Hypothesis 3 is not supported by evidence from clarification behavior.

### Respondents' engagement

To test our Hypotheses 4–6 about respondents' engagement, we first focus on respondents' gaze at the virtual interviewers, and then on their acknowledgment behaviors, smiles, and subjective assessments of the virtual interviewer.

#### Gaze at the virtual interviewer

**Figure 3:**
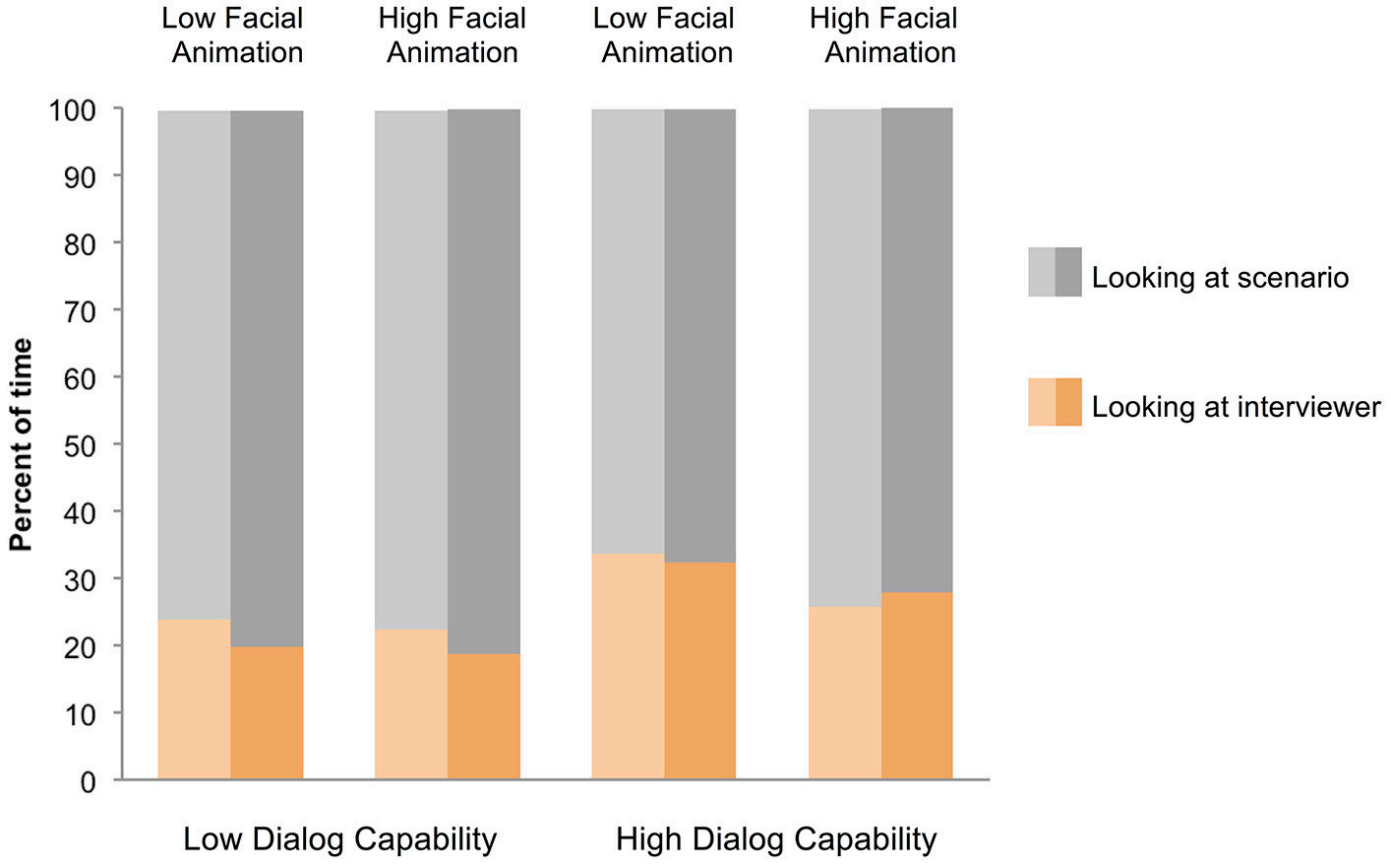
**Percentage of time that respondents looked at the virtual interviewer and the scenario packet, on average, across the four conditions, broken down by whether they were answering questions that mapped onto the scenario in a straightforward (lighter shades) or complicated (darker shades) way**. Gaze elsewhere (not at the virtual interviewer or scenario packet) was so rare (less than 1% of the time in all conditions) that it is not plotted.

From the video recordings of respondents' faces, we used Sequence Viewer (http://www.sequenceviewer.nl/) to code whether respondents were looking at the screen (i.e., at the virtual interviewer), at their paper packet, or elsewhere at every moment in each interview (from the research assistants' observations of video monitors during the pre-interview training sessions, we knew that respondents had all looked at the virtual interviewer for several minutes before the survey interview, as instructed). Respondents looked almost exclusively at their scenario packet and the virtual interviewer; they looked elsewhere in the room so rarely (less than 1% of the time) as to be negligible (see Figure 3).

Consistent with Hypothesis 4, respondents spent a greater proportion of the interview time looking at the high-dialog-capability virtual interviewers (29.8% of the time) than the low-dialog-capability virtual interviewers (21.1%), *F*_(1, 69)_ = 6.73, *p* = 0.012, η^2^ = 0.089. In order to further explore this phenomenon (that is, to further understand how respondents' engagement as measured by gaze connected with clarification dialog), we examined respondents' gaze at the virtual interviewers for complicated and straightforward scenarios, because it was only in complicated scenarios that clarification dialog ever occurred. As Figure 3 shows, respondents looked slightly but reliably *less* at the virtual interviewer (and more at their scenario packets) when the mappings between questions and scenarios were complicated (24.7% of the time) rather than straightforward (26.3% of the time), *F*_(1, 69)_ = 4.20, *p* < 0.05, η^2^ = 0.057. This overall difference resulted particularly from the low-dialog-capability conditions (19.2% of the time for complicated scenarios and 23.1% for straightforward) rather than the high-dialog-capability conditions, where there was no difference in the proportions of time spent looking at the virtual interviewer based on scenario mappings (30.1 vs. 29.6%), interaction *F*_(1, 69)_ = 7.16, *p* < 0.01, η^2^ = 0.094. Our interpretation is that in the low-dialog-capability conditions respondents were left to their own devices to figure out the right answer to the survey question, and so the only available useful information, if the virtual interviewer would not provide clarification, could come from examining the scenarios more closely. In the high dialog capability conditions, engagement with the virtual interviewer through gaze was greater and not related to the content of the scenarios^1^.

Contrary to Hypothesis 5, there is not sufficient evidence that respondents looked more at the virtual interviewers with high facial animation than those with low facial animation, *F*_(1, 69)_ = 1.22, *p* = 0.27, η^2^ = 0.017. An estimated Bayes_10_ factor (alternative/null) of 0.669 suggested that the data were 1.49:1 in favor of the null hypothesis, that is, 1.49 times more likely to occur under a model that does not include an effect of facial animation, rather than a model that does include it.

Contrary to Hypothesis 6, the virtual interviewer's facial animation did not interact with its dialog capability in affecting respondents' gaze behavior, *F*_(1, 69)_ = 0.50, *p* = 0.48, η^2^ = 0.017. An estimated Bayes_10_ factor (alternative/null) of 1.771 does not rule out the possibility that the data may favor Hypothesis 6, but it seems unlikely.

There are at least two possible explanations for this pattern of results—that gaze increased with high-dialog-capability but not high-facial-animation interviewers—given that our experimental conditions varied on more than one feature. One is that respondents with a high-dialog-capability virtual interviewer found the content of the interviewer's contributions (e.g., clarification dialog) compelling and human-like enough to spend a greater proportion of their time looking at the interviewer. Another is that respondents with a high-dialog-capability virtual interviewer fully trusted what they were told about the interviewer's perceptual capacity in the experiment instructions: that the high-dialog-capability interviewer could perceive their facial expression and gaze. The fact that respondents in the high-dialog-capability conditions were explicitly instructed to look at the interviewer when ready for the next question makes disentangling this more difficult, but we note that the increase in looking time at the high-dialog-capability interviewer is *proportional*, and occurs along with a substantial increase in interview duration; high-dialog-capability interviews took 7.26 min on average (SE 0.36 min) compared with low-dialog-capability interviews (5.53 min, SE 0.37 min), *F*_(1, 69)_ = 11.23, *p* = 0.001, η^2^ = 0.140. So the increase in looking time seems to us unlikely to result only from looking at the interviewer during transitions between survey questions, which would need to be quite long (a full minute of the interview, or a full 5 s at each question transition) to account for the effect.

Although respondents in this experiment did look at their paper packets a substantial proportion of the time during the interview (which means that at those moments they could only have been listening to—not watching—the virtual interviewer), we consider the proportions of time looking at the virtual interviewer observed here to be sufficient to allow us to detect potential effects of the virtual interviewer's facial animation even in the conditions with less looking time. The fact that we did observe significant differences in multiple measures based on facial animation corroborates this judgment.

#### Acknowledgment behaviors

In face-to-face interactions interlocutors can acknowledge each other's utterances verbally and visually: they can use back channel utterances (e.g., “okay,” “all right,” “got it,” “thank you”; Yngve, 1970) and they can nod, shake their heads, shrug their shoulders, raise their eyebrows, etc., in order to communicate continued attention and possible understanding (Allwood et al., 1992; McClave, 2000). Verbal and visual acknowledgments can be seen as part of an integrated multimodal system (Carter and Adolphs, 2008) that displays engagement in an interaction.

To examine acknowledgments in our virtual interviews, we counted respondents' verbal back channel utterances from the interactional moves we had coded (see Supplementary Table 4). We also coded head movements (nods, head shakes, other head movements like tilts), and other body or facial movements (like shoulder shrugs and eyebrow raising), using Sequence Viewer, based on the video recordings of the interviews. Just as reliability was measured for the interactional move coding (86.6% of question-answer sequences double-coded, see Section Comprehension), it was measured for these behaviors as well. Each of the individual behaviors was relatively rare in our sample, but coders' level of agreement was high: for head movements the coders' judgments agreed 92.5% of the time, and for other body movements they agreed 94.1% of the time [Cohen's kappas for these reliabilities were low, at 0.32 and 0.27, but as Viera and Garrett (2005) demonstrate, kappa can easily be a misleading index of agreement when the occurrence of what is coded is rare].

In our tests of Hypotheses 4–6, we first looked at verbal backchannels alone, head movements alone, and particular body and facial movements alone. Because backchannels and particular head movements and particular body and facial movements occurred rarely enough that there was a risk that we would miss patterns relevant to our hypotheses given our sample size, we also aggregated across nods, head shakes, other head movements, and other body and facial movements.

For Hypothesis 4 (effects of interviewer's dialog capability on respondent engagement), we see only suggestive evidence in support of it. Respondents did not produce many backchannels (and many produced none), but they produced marginally more of them with the high dialog capability agents (0.32 per interview) than with the low-dialog-capability agents (0.18 per interview), *F*_(1, 69)_ = 2.82, *p* = 0.098, η^2^ = 0.039. Analyses of all facial and bodily movements do not show any significant effects.

**Figure 4:**
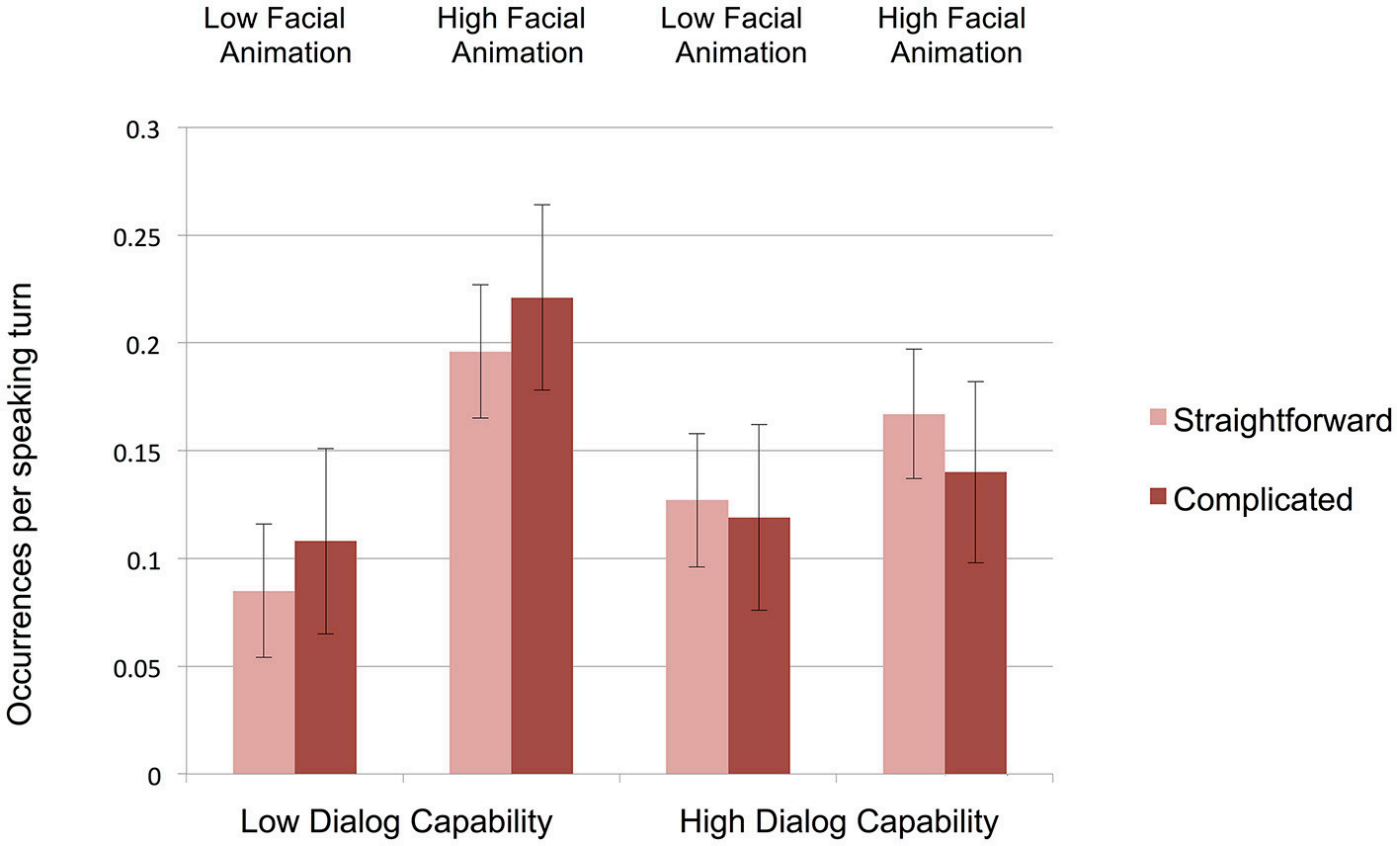
**Respondents' rates of aggregated acknowledgment behaviors (verbal back channels, nods, head shakes, other head movements, and other body and facial movements) per speaking turn (error bars represent SE's)**.

The evidence for Hypothesis 5 (effects of interviewer facial animation on respondent engagement) is also suggestive. Respondents were marginally more likely to produce one of these movements when the virtual interviewer had high facial animation (averaging 0.13 occurrences per speaking turn) than when virtual interviewer had low facial animation (0.08 occurrences per speaking turn, *F*_(1, 69)_ = 3.21, *p* = 0.078, η^2^ = 0.039. But support for Hypothesis 5 becomes stronger if we also include verbal back channel utterances, taking Carter and Adolphs' (2008) multimodal view of acknowledgment behavior. As Figure 4 shows, respondents were nearly twice as likely to display our aggregated acknowledgment behaviors (visual and verbal) when the virtual interviewer had high facial animation (at a rate of 0.18 occurrences per speaking turn) than when the virtual interview had low facial animation (0.11 occurrences per speaking turn), *F*_(1, 69)_ = 4.29, *p* < 0.05, η^2^ = 0.059.

Hypothesis 6 predicted an interaction of the form that respondents would produce disproportionately more engagement behaviors with high-dialog-capability high-facial-animation virtual interviewers, and proportionately fewer with low-dialog-capability low-facial-animation interviewers. We see partial evidence in support of this hypothesis in one significant interaction of interviewer dialog capability and facial animation with respect to nods, *F*_(1, 69)_ = 5.81, *p* = 0.019, η^2^ = 0.078. Partially consistent with Hypothesis 6, respondents nodded least with the low-dialog capability low-facial-animation interviewer (0.05 times per interview), but (unexpectedly) most with the high-dialog-capability *low*-facial animation-interviewer (0.26 times per interview). There were no other significant interaction effects.

#### Smiles

Another measure of respondents' engagement with the virtual interviewers is their frequency of smiling.

We thus coded respondents' smiles in order to compute smile frequency and duration. The coder (one of the authors) had been certified in the Facial Action Coding System (FACS; Ekman and Friesen, 1978). We determined coding reliability for all the question-answer sequences for a subsample of 20% of the respondents, equally distributed in the four experimental conditions, as independently coded by a second coder (four respondents had to be excluded from this analysis because the resolution of the video was not sufficient for this level of facial coding). Coders' level of agreement on smile frequency was high (92.1%), with a Cohen's kappa of 0.66. Coders' judgments on smile duration were also highly correlated, *r* = 0.835, *p* < 0.0001 (considering all sequences) and *r* = 0.762, *p* < 0.0001 (considering only those sequences in which at least one smile was found by at least one coder).

Regarding Hypothesis 4, there were no reliable effects of the interviewer's dialog capability on smiles.

Regarding Hypothesis 5, respondents interacting with a high facial animation virtual interviewer smiled marginally more often (2.25 times over the course of their interview, SE 0.55) than respondents interacting with a low facial animation virtual interviewer (0.78 times, SE 0.55), *F*_(1, 68)_ = 3.62, *p* = 0.061, η^2^ = 0.050. Respondents interacting with a high facial animation virtual interviewer also smiled marginally longer (11.5 s over the course of the interview, SE 3.1) than respondents interacting with a low facial animation virtual interviewer (3.0 s, SE 3.1), *F*_(1, 68)_ = 3.67, *p* = 0.060, η^2^ = 0.051.

Regarding Hypothesis 6, there were no significant interactive effects of virtual interviewers' dialog capability and facial animation on respondents' smiles.

#### Respondents' self-reported subjective experience

A final set of measures of respondents' engagements was their responses to the post-experiment questionnaire in which they reported how they felt about and evaluated the virtual interviewers.

Table 5 presents the average ratings as well as ANOVA statistics for tests of Hypotheses 4–6^2^. Note that all of these ratings are lower than one would expect if the respondents evaluated the virtual interviewer as being very human-like. But given the constraints of a standardized interviewing situation, it is also plausible that human interviewers who implemented these interviews would not be rated as particularly autonomous, personal, close, or sensitive, and they might also be rated as more robotic than human (the term “robotic” is sometimes used to caricature the behavior of rigidly standardized interviewers, for example in survey invitations, see Conrad et al., 2013).

**Table 5:** **Respondents' subjective ratings of the virtual interviewers, presented in the order the ratings were elicited (SE's in parentheses)**.

	**Response options**	**Low dialog capability**		**High dialog capability**		**Test of hypothesis 4: effect of dialog capability**	**Test of hypothesis 5: effect of facial animation**	**Test of hypothesis 6: interaction of dialog capability and facial animation**
		**Low facial animation**	**High facial animation**	**Low facial animation**	**High facial animation**			
How comfortable were you with Derek at the start of the session?	1: Not at all comfortable 5: Very comfortable	3.83 (0.27)	3.22 (0.27)	3.33 (0.27)	3.00 (0.26)	*F*_(1, 69)_ = 1.83, *p* = 0.180, η^2^ = 0.026	***F*_(1, 69)_ = 3.13**, ***p* = 0.081**, **η^2^ = 0.043**	*F*_(1, 69)_ = 0.27, *p* = 0.604, η^2^ = 0.004
As the interview progressed, did your comfort with Derek increase, decrease, or stay the same?	1: Decrease 2: Stay the same 3: Increase	1.78 (0.15)	1.78 (0.15)	1.56 (0.15)	1.21 (0.15)	***F*_(1, 69)_ = 7.05,** ***p* = 0.010,** **η^2^ = 0.093**	*F*_(1, 69)_ = 1.35, *p* = 0.250, η^2^ = 0.019	*F*_(1, 69)_ = 1.35, *p* = 0.250, η^2^ = 0.019
How natural was the interaction with Derek?	1: Not at all natural 5: Very natural	3.22 (0.27)	2.61 (0.27)	3.17 (0.27)	2.74 (0.27)	*F*_(1, 69)_ = 0.02, *p* = 0.898, η^2^ = 0.000	***F*_(1, 69)_ = 3.65,** ***p* = 0.06,** **η^2^ = 0.050**	*F*_(1, 69)_ = 0.11, *p* = 0.741, η^2^ = 0.002
How often did Derek seem to act on his own?	1: Never 5: All the time	2.56 (0.25)	2.44 (0.25)	3.83 (0.25)	2.74 (0.25)	***F*_(1, 69)_ = 9.82,** ***p* = 0.003,** **η^2^ = 0.124**	***F*_(1, 69)_ = 5.80,** ***p* = 0.019,** **η^2^ = 0.078**	***F*_(1, 69)_ = 3.86,** ***p* = 0.053,** **η^2^ = 0.053**
Would you say that Derek acted more like a computer or a person?	1: Just like a computer 2: As much like a computer as a person 3: Just like a person	1.44 (0.152)	1.61 (0.15)	2.11 (0.15)	1.68 (0.15)	***F*_(1, 69)_ = 6.02,** ***p* = 0.017,** **η^2^ = 0.080**	*F*_(1, 69)_ = 0.75, *p* = 0.391, η^2^ = 0.011	***F*_(1, 69)_ = 3.88,** ***p* = 0.053,** **η^2^ = 0.053**
How much did you enjoy interacting with Derek?	1: Did not enjoy at all 5: Thoroughly enjoyed	3.06 (0.22)	3.22 (0.22)	3.83 (0.22)	3.37 (0.22)	***F*_(1, 69)_ = 4.41,** ***p* = 0.039,** **η^2^ = 0.060**	*F*_(1, 69)_ = 0.46, *p* = 0.500, η^2^ = 0.007	*F*_(1, 69)_ = 2.06, *p* = 0.156, η^2^ = 0.029
How frustrating was it to be interviewed by Derek?	1: Not at all frustrating 5: Very frustrating	1.88 (0.24)	2.11 (0.23)	1.50 (0.23)	1.84 (0.22)	*F*_(1, 68)_ = 2.01, *p* = 0.161, η^2^ = 0.029	*F*_(1, 68)_ = 1.54, *p* = 0.219, η^2^ = 0.022	*F*_(1, 68)_ = 0.06, *p* = 0.806, η^2^ = 0.001
I felt that Derek was…	1: Impersonal 5: Personal	1.94 (0.23)	2.35 (0.24)	3.28 (0.23)	2.68 (0.23)	***F*_(1, 68)_ = 12.72,** ***p* = 0.001,** **η^2^ = 0.158**	*F*_(1, 68)_ = 0.16, *p* = 0.693, η^2^ = 0.002	***F*_(1, 68)_ = 4.61,** ***p* = 0.035,** **η^2^ = 0.063**
I felt that Derek was…	1: Distant 5: Close	2.17 (0.24)	2.77 (0.25)	3.19 (0.26)	2.79 (0.24)	***F*_(1, 66)_ = 4.55,** ***p* = 0.037,** **η^2^ = 0.064**	*F*_(1, 66)_ = 0.17, *p* = 0.685, η^2^ = 0.003	***F*_(1, 66)_ = 4.13,** ***p* = 0.046,** **η^2^ = 0.059**
I felt that Derek was…	1: Inexpressive 5: Expressive	2.56 (0.29)	2.78 (0.29)	3.00 (0.29)	2.84 (0.28)	*F*_(1, 68)_ = 0.79, *p* = 0.377, η^2^ = 0.011	*F*_(1, 68)_ = 0.01, *p* = 0.911, η^2^ = 0.000	*F*_(1, 68)_ = 0.44, *p* = 0.509, η^2^ = 0.006
I felt that Derek was…	1: Insensitive 5: Sensitive	2.56 (0.22)	2.94 (0.22)	3.22 (0.22)	3.32 (0.22)	***F*_(1, 69)_ = 5.59,** ***p* = 0.021,** **η^2^ = 0.075**	*F*_(1, 69)_ = 1.21, *p* = 0.275, η^2^ = 0.017	*F*_(1, 69)_ = 0.45, *p* = 0.503, η^2^ = 0.007

*Statistically significant and marginal effects are in bold*.

As detailed in Table 5, Hypothesis 4 is supported on several fronts. Respondents with an interviewer high in dialog capability reported enjoying the interview more, and they rated the interviewer as more autonomous, more personal, less distant, and more sensitive than respondents with an interviewer low in dialog capability^3^. They also rated the interviewer as less like a computer. Unexpectedly, respondents with high dialog capability interviewers reported a greater decrease in comfort across the interview than respondents with the low dialog capability interviewers.

In contrast to the predictions of Hypothesis 5, there were significant effects of facial animation suggesting that interviewers with *low* facial animation were in some ways preferred. Respondents with low facial animation interviewers reported marginally greater comfort with the interviewer at the start of the session, and they rated the interviewer as marginally more natural and as reliably more autonomous (acting on his own), than did respondents with high facial animation interviewers.

The pattern uncovered in tests of Hypothesis 6 is consistent with that found for acknowledgments. Respondents with low facial animation interviewers were more likely (albeit marginally) to rate the interviewer as autonomous when the interviewer had high dialog capability (see Table 5). These same respondents were also particularly more likely to rate the low facial animation interviewer as more personal, as less distant (closer), and as marginally more like a person than a computer. In other words, respondents found the interviewer to be particularly autonomous and personal when he looked more robotic (displayed less facial movement) but could converse like a human. The fact that the mean ratings in this condition (low facial animation/high dialog capability) stand out from the others, along with the (marginal) interaction effects, suggests that part of what is driving the main effects of dialog capability and facial animation on these items are the perceptions of this subgroup.

## Discussion

### Summary

The findings reported here document that two important elements of human face-to-face interaction—dialog capability and facial movement—implemented in virtual survey interviewers differently affect respondents' comprehension and the nature of their engagement with the virtual interviewer. As tested in Hypotheses 1 and 4, respondents who interacted with a virtual interviewer with greater dialog capability (that is, which could help respondents interpret the questions as intended) provided more accurate answers and took more responsibility for their comprehension, requesting clarification more often. They looked at high-dialog-capability interviewers more, they produced marginally more backchannel responses, and they reported enjoying the interview more and finding the interviewer to be more personal and less distant. As tested in Hypotheses 2 and 5, respondents who interacted with a virtual interviewer with more facial animation displayed more evidence of engagement—more verbal back channels and visual acknowledgments of the interviewer's utterances, and marginally more smiles. They also reported *less* comfort with the high facial animation interviewers and rated these interviewers as less natural. In testing Hypotheses 3 and 6, we observed that respondents (unexpectedly) nodded more and rated the virtual interviewer as more personal and less distant if it had high dialog capability and *low* facial animation.

The current findings extend work on people's reactions and behaviors when they talk with interviewing agents, for example telling stories to an agent that exhibits listening behavior (e.g., Gratch et al., 2006; von der Pütten et al., 2010), answering open-ended questions asked by a peer (e.g., Bailenson et al., 2006) or answering open-ended questions asked by a deception-detecting kiosk agent (Nunamaker et al., 2011), to the task of a survey interview for social measurement that uses closed categories as response options and that is designed to make statistical estimates of a population. The findings also extend work on disclosure of sensitive information in a survey interview with a virtual interviewer (Lind et al., 2013) to an interview with non-sensitive questions that have verifiably correct and incorrect answers, and in which accurate comprehension of the terms in the questions is critical. Because of the nature of this survey task, our measures focus on aspects of the interaction and of respondents' behavior (e.g., response accuracy, smiles, acknowledgments) that have not been the focus in previous studies, where users' nuanced interpretation of what the virtual interviewer is asking is less essential.

While it is unclear where exactly our survey task fits into a taxonomy of tasks for which virtual humans have been designed, what is clear is that for this task the two features we experimentally manipulated have quite distinct effects. We assume this is because they engage different channels of communication (the exchange of spoken vs. visual information) and manifest themselves over different time scales—a virtual agent's facial animation is visible to users as soon as any talking starts, while evidence of the agent's dialog capability unfolds more incrementally over time as the interviewer does or does not respond to the user's need for clarification. We hypothesize that our findings should generalize to other interactive tasks with virtual agents that share the central features of the current task: a need for grounding interpretation of terms in an agent's utterances and a need for the user to be sufficiently engaged to complete a task that someone else has initiated (Schober et al., 2003).

While our experimental design allows us to see effects of what we manipulated, it does not allow us to disentangle the relative contributions of the bundled features that comprise the different agents. Of course, our agents' particular features could have been implemented differently (e.g., the agents could have had different vocal or visual attributes, or been unnamed or have had different names), and it is unknown how our findings would generalize to different implementations. Our experimental design also does not allow inference about potential (and intriguing) causal connections between our different measures. For example, we do not know whether respondents' attributions about the high-dialog-capability interviewer *result from* or *cause* or are *independent of* their improved comprehension: did respondents answer more accurately with a high dialog capability virtual interviewer because they enjoyed the interview more and found the interviewer more perceptive and responsive? Or did they enjoy the interview more because they were confident that they had comprehended the questions as intended? Did respondents smile more often and longer with a high facial animation virtual interviewer because they felt more engaged, as one might expect given Krämer et al.'s (2013) finding that users who were engaged in small talk with a virtual agent smiled more when the virtual agent smiled more? Or, alternatively (and consistent with our respondents' reports of less comfort), did they smile more because their smiles reflected distress or discomfort (e.g., Ansfield, 2007)? The fact that respondents' subjective experience of a virtual survey interviewer—their level of comfort, their enjoyment, how natural they feel the interaction to be—can be correlated with their disclosure of sensitive information (Lind et al., 2013) makes it plausible that users' affective reactions could be causally connected with their comprehension and behavioral displays even with non-sensitive survey questions of the sort asked here, but the current data only allow speculation.

### Designing virtual survey interviewers

Animating virtual interviewing agents that could be used in, for example, a web survey with textual response is becoming increasingly straightforward with off-the-shelf tools. Instantiating dialog capability and speech recognition is a greater challenge, but the constrained nature of the survey interview task (a finite set of possible turns that can occur, standardized wording of questions, closed response options with limited vocabulary that a speech recognition system can handle, definitions of key terms already existing) can make implementing clarification dialog in a textual or speech interviewing system more plausible than in more open-ended or free-form conversational domains (Johnston et al., 2013; Schober et al., 2015).

Given the many possible ways to instantiate a virtual interviewer—a range of possible expressivity, sensing capabilities and responsiveness to respondents' signals, and a range of more and less human-like facial motion and detail—we propose the following design considerations for building virtual interviewers for actual surveys that produce population estimates:

- *Designing to maximize participation:* Potential respondents are likely to vary in whether they will consent to interact with a virtual interviewer, for example, in an online survey. Perhaps the greatest deterrent is uncanniness (e.g., MacDorman et al., 2009). The fact that participants in the current study reported that the virtual interviewers with more facial animation made them less comfortable and were less natural than virtual interviewers with less facial movement could result from people's finding the increased realism of high facial animation to be eerie, and this might reduce participation in virtual interviews by some sample members. But for others, this might not affect participation; in the Lind et al. (2013) study with a more cartoon-like interviewer, different respondents had completely opposite affective reactions from each other to the very same interviewing agent, and this correlated with their willingness to disclose sensitive information.
- *Designing to maximize completion:* Although in this study we did not include an interviewing condition without a facial representation, the increased engagement (more acknowledgments and smiles) that we observed with the high facial animation interviewers could translate to increased completion of questionnaires compared to self-administered online questionnaires without any virtual interviewer. Engagement could promote completion if respondents apply social norms from face-to-face interaction in which it would be rude to break off a conversation midstream, or because a moving talking face simply makes the task more interesting. To investigate this, one would need to carry out a study outside the laboratory (e.g., online) with naturalistic incentives (rather than our laboratory method with payment).
- *Designing to maximize comprehension:* As we have proposed for human interviewers (Schober and Conrad, 1997; Conrad and Schober, 2000), enabling virtual survey interviewers to engage in clarification dialog is likely to improve respondents' understanding of questions and thus the quality of the data collected in the survey. There are a number of ways to instantiate clarification dialog in a virtual interviewer, from providing scripted (spoken or even textual) definitions only when respondents request them to diagnosing the potential need for clarification based on respondents' disfluencies and gaze aversion (e.g., Ehlen et al., 2007; Schober et al., 2012). The findings in the current study suggest that system-initiated clarification is likely to be important for maximizing comprehension.
- *Designing the interviewer's appearance and voice:* It is essentially impossible to design a virtual human interviewer without creating the perception of some demographic characteristics. If the virtual interviewer communicates by speaking, its speech will inevitably have attributes such as dialect, a pitch range, prosody, and vocal quality. How the current findings, which are based on one 3D head model with particular visual and linguistic attributes, will generalize to virtual interviewers with other visual and linguistic attributes, will be a key design question: how a virtual interviewer's visual attributes (skin shade, eye color, hair style, facial features, clothing, hair covering, etc.) or speech style (accent, vocabulary, pronunciation) will affect respondents' judgments about the interviewer's perceived “social identity” (gender, race, social class, education, religious affiliation) and potentially respondents' answers to questions on some interview topics. It is well known that demographic characteristics of human interviewers can (undesirably) affect the distribution of responses (e.g., Hatchett and Schuman, 1975) even in telephone interviews where only voice attributes are available (e.g., Cotter et al., 1982; Finkel et al., 1991). There is preliminary evidence that this kind of interviewer effect may also appear with virtual interviewers (Conrad et al., 2011), and that gender and nationality attributions can occur for embodied agents more generally (Eyssel and Hegel, 2012; Eyssel and Kuchenbrandt, 2012).
- *Designing for different types of survey questions*: The current research suggests that virtual interviewers implemented with high dialog capability may promote accurate answers to factual questions about mundane topics for which complicated mappings are possible. However, it has been shown (Lind et al., 2013) that when virtual interviewers ask questions about sensitive topics, respondents seem to answer most questions less truthfully (disclose less sensitive information) than when the same questions are spoken by a disembodied (audio) interviewer. If a survey investigates both non-sensitive and sensitive topics, one could imagine implementing the virtual interviewer for only the non-sensitive questions. To our knowledge this has never been attempted; much is unknown about how the intermittent display of a virtual interviewer might affect respondents' affective responses and whether removing an interviewer—after being present—could convincingly create a sense of privacy.
- *Giving respondents a choice of interviewer?* One potential advantage of implementing virtual survey interviewers is that one could let *respondents* choose an interviewer with the attributes (appearance, speech style) that they prefer, which is not a possibility with human interviewers. It is entirely unknown which attributes respondents would most want to be able to choose, whether providing choices will increase respondents' engagement and data quality, or how choosing an interviewer that makes respondents most comfortable might affect their effort in producing accurate responses.

Considering factors such as these, as well as those raised by Cassell and Miller (2008), will be essential if virtual survey interviewing systems are to be effective. The need for accurate survey data will continue; the question will be what kinds of interviewers and interviewing systems will best promote accurate data and respondent engagement in new technological environments (Schober and Conrad, 2008), and what role embodied interviewing agents might best play.

### Conflict of interest statement

The authors declare that the research was conducted in the absence of any commercial or financial relationships that could be construed as a potential conflict of interest.

## Footnotes

^1^This pattern of findings rules out an interpretation that greater gaze at the high-dialog-capability virtual interviewer resulted simply from respondents looking at the interviewer more at turn transitions, which is a well-documented phenomenon in human dialog (e.g., Kendon, 1967). If this were the case, then there should be more looking at the interviewer for complicated than straightforward scenarios with the high dialog capability interviewers, because complicated scenarios involved more transitions (because of more clarification).

^2^We report parametric statistical analyses on these rating scale data so as to straightforwardly test our hypotheses about our two experimental factors and potential interactions. This approach is supported by Norman's (2010) arguments and evidence about the robustness of parametric analyses for interval data. But this does not mean that we are claiming that our participants treated the distances between intervals on our scales as equal, which is, of course, unknowable (Jamieson, 2004); we simply are claiming that higher ratings are higher.

^3^We interpret the finding that autonomy was rated as lower for low-dialog-capability agents as reflecting respondents' assessment of the virtual interviewer's ability to reason and think (“act on his own”), as opposed to reflecting a judgment that the virtual interviewer had a human operator rather than being stand-alone software. While we can't, of course, rule out this possibility, the fact that the patterns of ratings are consistent on more items beyond the question about autonomy supports this view.
