# Supplementary material for: Comprehension and engagement in survey interviews with virtual agents
Source: Front Psychol. 2015 Oct 20;6:1578. doi: 10.3389/fpsyg.2015.01578 (PMC4611966; doi:10.3389/fpsyg.2015.01578)
Supplement: Supplementary file 1 [file Table1.PDF]

Supplementary Table 1 | Survey questions and definitions. Key concepts in the questions that have definitions from the US Bureau of the Census and/or the US Bureau of Labor Statistics are italicized.

**SURVEY QUESTIONS**	
Housing question 1	How many *bedrooms* are there in this house?
Housing question 2.1*	This question has two parts. How many *full bathrooms* are there in this house?
Housing question 2.2*	Thanks. How many *half bathrooms* are there?
Housing question 3	How many *other rooms* are there, other than bedrooms and bathrooms?
Housing question 4	How many people *live in this house*?
Employment question 1	Does anyone in this household have a *business* or a farm?
Employment question 2	Last week, did Chris do any *work for pay*?
Employment question 3	Last week, did Pat have *more than one job*, including part-time, evening or weekend work?
Employment question 4	How many hours per week does Mindy *usually* work at her job?
Purchases question 1	Has Carla purchased or had expenses for *car tires*?
Purchases question 2	Has Alexander purchased or had expenses for *college tuition or fixed fees*?
Purchases question 3	Has Kelly purchased or had expenses for *household furniture*?
Purchases question 4	Has Dana purchased or had expenses for *meats and poultry*?

**DEFINITIONS**	
Bedroom<br>(Housing question 1)	A bedroom is a finished room specifically designed by the owner to be used for sleeping. A bedroom does NOT have to be used for sleeping in order to qualify as a bedroom. For example, a bedroom that is being used as an office should be counted as a bedroom.<br><br>Do NOT count as a bedroom any room that was designed for another purpose but is being used as a bedroom. For example, a den being used as a bedroom is still a den and should not be counted as a bedroom.<br><br>Do NOT count as a bedroom any dens, living rooms, or other rooms that can be converted at night for sleeping.<br><br>Do NOT count any bedroom that the renter is denied access to or use of by the owner.<br><br>A one-room efficiency apartment does not have a bedroom.
Full and half bathrooms<br>(Housing questions 2.1 and 2.2)	A full bathroom has (1) a flush toilet, (2) a bathtub or shower, and (3) a sink or washbasin with running water. Bathrooms that contain all of the above items, whether separated by a partition or door, are to be considered a full bathroom.

	A half bathroom has any two of these three items: (1) a flush toilet, (2) a bathtub or shower, and (3) a sink or washbasin with running water.<br><br>If the only bathroom facilities do not meet the definition of a full or half bath, answer zero. (For example, if there is only a flush toilet in a room).<br><br>If a bathroom is shared by the occupants of more than one housing unit, the bathroom is included with the unit from which it is most easily reached.
Other rooms (Housing question 3)	Include whole rooms such as living rooms, dining rooms, kitchens, lodger's rooms, finished basements or attic rooms, recreation rooms, and permanently enclosed sun porches. Rooms used for offices by a person living in the unit are also included in this survey. Rooms are counted even if they are not used.<br><br>Do NOT include bedrooms, bathrooms, unfinished attics or basements, halls, foyers or vestibules, balconies, closets, alcoves, pantries, strip or pullman kitchens, laundry or furnace rooms, open porches, and unfinished spaces used for storage.<br><br>A partially divided room, such as a dinette next to a kitchen or living room, is a separate room ONLY if there is a PERMANENT PARTITION FROM FLOOR TO CEILING BETWEEN THE TWO AREAS. An L-shaped room, a "great" room, or a step-down is therefore counted as one room unless there is a permanent partition dividing the room into parts.<br><br>If a room is used by occupants of more than one unit, the room is included with the unit from which it is most easily reached.<br><br>Do NOT count any rooms that the renter is denied access to or use of by the owner. Do count rooms REGARDLESS of their year-round usability.<br><br>Exclude all bathrooms. While some rooms, such as a small room with only a wash basin, do not meet the definition of a bathroom, they are also to be excluded from the count of other rooms.
Living in a housing unit (Housing question 4)	A person is considered to be living in a housing unit even if the person is not present at the time of the survey. Live-in servants or other employees, lodgers, and members of the household temporarily away from the unit on business or vacation are included in the count.<br><br>Do NOT count any people who would normally consider this their (legal) address but who are LIVING away on business, in the armed

	forces, or attending school (such as boarding school or college).<br><br>Do NOT count overnight lodgers, guests and visitors. Do NOT count day employees who live elsewhere.
Business<br>(Employment question 1)	A business exists when one or more of the following conditions is met: Machinery or equipment of substantial value is used in conducting the business, or an office, store, or other place of business is maintained, or the business is advertised by: listing in the classified section of the telephone book, or displaying a sign, or distributing cards or leaflets or otherwise publicizing that the work or service is offered to the general public.
Work for pay<br>(Employment question 2)	Include piece rate income as earnings. Persons working in garment making or food packaging often receive this type of income. Also count college assistantships and fellowships and on the job training as earnings.<br><br>DO NOT INCLUDE PAY IN KIND, such as food or lodging for work, or expense accounts as earnings.
More than one job<br>(Employment question 3)	A job exists when there is a definite arrangement for regular work every week, or every month, for pay or other compensation (such as profits, anticipated profits, or pay in kind, such as room and board). A formal, definite arrangement with one or more employers to work on a continuing basis for a specified number of hours per week or days per month, but on an irregular schedule during the week or month, is also a job.<br><br>It is possible for individuals to have more than one employer, but only one job. If an individual does the same type of work for more than one employer in an occupation where it is common to have more than one employer, do not consider the individual a multiple jobholder. Examples include private household or domestic workers including babysitters, chauffeurs, gardeners, handypersons, cooks, and maids.
Usually<br>(Employment question 4)	By usually, we mean 50% of the time or more, or the most frequent schedule during the past 4 or 5 months.
Car tires<br>(Purchases question 1)	Consider new, recapped, or retreaded tires for automobiles. Do not include tires for vans and trucks.
College tuition or fixed fees<br>(Purchases question 2)	Consider tuition and fixed fees paid to public or private institutions offering credit beyond the high school level. Do not include payments to vocationally oriented schools such as business, technical, trade, or secretarial; do not include payments for room and board, books, lab fees, etc.

Household furniture (Purchases question 3)	By furniture, we mean tables, chairs, footstools, sofas, china cabinets, utility carts, bars, room dividers, bookcases, desks, beds, mattresses, box springs, chests of drawers, night tables, wardrobes, and unfinished furniture. Do not include TV, radio, and other sound equipment, lamps and lighting fixtures, outdoor furniture, infants' furniture, or appliances.
Meats and poultry (Purchases question 4)	Please include beef, lamb, pork, game; organ meats, such as kidneys, sweetbreads, chitterlings, heart, tongue; sausages and luncheon meats; poultry, such as chicken, turkey, pheasant, goose, and duck. Include canned ham. Do not include other canned meats and canned poultry, or any prepared meats and poultry.

* Housing Question 2 has two parts, one about full bathrooms and one about half bathrooms; the half bathroom part of the question was what was evaluated in all analyses reported here.
