# Supplementary material for: Comprehension and engagement in survey interviews with virtual agents
Source: Front Psychol. 2015 Oct 20;6:1578. doi: 10.3389/fpsyg.2015.01578 (PMC4611966; doi:10.3389/fpsyg.2015.01578)
Supplement: Supplementary file 2 [file Table2.PDF]

Supplementary Table 2 | Additional recorded interviewer utterances.

**Interview introductions**	
Introduction 1	Hello. My name is Derek, and today I'll be asking you a few questions about housing, jobs, and purchases. Before we begin, I'd like to confirm that you have the right packet. Please tell me your packet number.
Introduction 2	Let's get started!<br><br>I'll be asking you to answer 12 survey questions, each about a different fictional scenario described in the packet. For the first question, you should use the information on the first page. For the second question, use the information on the second page, and so on.
Introduction 3	A page may contain a very short story about someone's living or working situation, a floor plan of a house or apartment, or a receipt from a purchase. You don't need to memorize the information on each page. Yet, you should be familiar with the information on each page, and you should answer the question based on that information. So, you should have the packet open to the page that corresponds to the question being asked. Before I ask the next question, I will instruct you to turn to the next page of your packet.
Introduction 4	The questions come from several different surveys conducted by the Bureau of Labor Statistics. According to their definitions, there is a correct answer for each question, and it is very important that you try hard to answer accurately. Knowing the correct answer depends on paying attention to the particular details of what is mentioned on each page of your packet. For example, imagine you're asked whether any power tools were purchased and on the imaginary page no power tools are mentioned, then you should answer "No."<br><br>Sometimes these survey questions use ordinary words with slightly different meanings than what you might be used to. Surveys often have technical definitions that are different from ordinary definitions.
Introduction 5	It is VERY important that you fully understand these instructions. If you have ANY questions, please ask them now.
Introduction 6	Go ahead and please begin familiarizing yourself with the scenarios.
High-dialog-capability additional introduction 1	For each question, I can provide definitions for key words. For instance, if the question asks about the number of alcoholic beverages purchased in a month, I can provide our definition of alcoholic beverages.

High-dialog-capability additional introduction 2	You shouldn't feel reluctant to ask me if you are at all unsure about what is meant by a perfectly ordinary word because I am looking for correct answers that fit our definitions most closely. In fact, I WANT you to ask if you have ANY uncertainty about how to interpret the question--even if this feels silly to you. I'll also try to help if it looks or sounds like you're having trouble. I will be more than happy to help you as much as possible.
**Pre-interview practice**	
Pre-interview transition 1	Are you ready to begin?
Pre-interview transition 2	Now, I'd like to walk you through a practice question to show you what the actual interview will be like. Please turn to the Practice Question.<br><br>First I will ask a question, and then you should respond according to the information in the scenario. Okay?
Practice question	"Did Gina buy any fats or oils?"
High-dialog-capability additional instruction	Before answering, you may need to ask me for a definition. Let's practice that. Please ask me if butter counts as a fat or oil.
High-dialog-capability additional definition	Margarine or butter is not considered to be a fat or oil.
High-dialog-capability additional instruction 2	With this information, you should be able to answer the question correctly. Please go ahead and tell me whether Gina bought any fats or oils.
Pre-interview conclusion	Thanks. That's the end of our practice.
**Neutral probes**	
Neutral probe 1	What is your best estimate?
Neutral probe 2	Is that a yes or a no?
Neutral probe 3	I'm sorry, but I need you to choose one number.
Neutral probe 4	Let me repeat the question.
Neutral probe 5	Whatever it means to you.
Neutral probe 6	I can't give you any more information about this question.
**Partial definitions (high-dialog capability interviewers)**	
Bedroom (Housing question 1)	Do NOT count as a bedroom any room that was designed for another purpose but is being used as a bedroom. For example, a den being used as a bedroom is still a den and should not be counted as a bedroom.
Full bathroom (Housing questions 2.1)	A full bathroom has (1) a flush toilet, (2) a bathtub or shower, and (3) a sink or washbasin with running water. Bathrooms that contain all of the above items, whether separated by a partition or door, are to be considered a full bathroom.

Half bathroom (Housing question 2.2)	A half bathroom has any two of these three items: (1) a flush toilet, (2) a bathtub or shower, and (3) a sink or washbasin with running water.
Other rooms (Housing question 3)	A partially divided room, such as a dinette next to a kitchen or living room, is a separate room ONLY if there is a PERMANENT PARTITION FROM FLOOR TO CEILING BETWEEN THE TWO AREAS. An L-shaped room, a "great" room, or a step-down is therefore counted as one room unless there is a permanent partition dividing the room into parts.
Living in a housing unit (Housing question 4)	Do NOT count any people who would normally consider this their (legal) address but who are LIVING away on business, in the armed forces, or attending school (such as boarding school or college).
Business (Employment question 1)	A business exists when one or more of the following conditions is met: Machinery or equipment of substantial value is used in conducting the business, or an office, store, or other place of business is maintained, or the business is advertised by: listing in the classified section of the telephone book, or displaying a sign, or distributing cards or leaflets or otherwise publicizing that the work or service is offered to the general public.
Work for pay (Employment question 2)	Count college assistantships and fellowships and on the job training as earnings.
More than one job (Employment question 3)	It is possible for individuals to have more than one employer, but only one job. If an individual does the same type of work for more than one employer in an occupation where it is common to have more than one employer, do not consider the individual a multiple jobholder. Examples include private household or domestic workers including babysitters, chauffeurs, gardeners, handypersons, cooks, and maids.
Usually (Employment question 4)	By usually, we mean 50% of the time or more, or the most frequent schedule during the past 4 or 5 months.
Car tires (Purchases question 1)	Do not include tires for vans and trucks.
College tuition or fixed fees (Purchases question 2)	Do not include payments to vocationally oriented schools such as business, technical, trade, or secretarial.
Household furniture (Purchases question 3)	Do not include TV, radio, and other sound equipment, lamps and lighting fixtures, outdoor furniture, infants’ furniture, or appliances.
Meats and poultry (Purchases question 4)	Do not include canned meats and canned poultry, or any prepared meats and poultry.

**Clarification offers**	
Clarification offer sounds	It sounds like you're having some trouble. Can I give you a definition that might help?
Clarification offer looks	It looks like you're having some trouble. Can I give you a definition that might help?
General help offer	Can I help you?
Specific help	What can I help you with?

**Dialog management**	
Yes	Yes
No	No
Okay	Okay
Thanks	Thanks.
Helpful	Thanks, that's helpful.
Wait	Please wait one moment.
Sorry	Sorry, let me repeat that.
Repeat	I'm sorry. I missed that. Can you repeat what you said?
No information	I'm sorry; I do not have that information.
Unable to comply	I'm sorry, but I can't do that.
Don't understand	I don't understand what you mean.

**Experiment administration**	
Housing transition	Now I'd like to ask you a few questions about housing.
Employment transition	Now I'd like to ask you a few questions about work.
Purchases transition	Now I'd like to ask you a few questions about purchases.
Next page 1	Thanks. Please turn to the next page of your packet.
Next page 2	Thank you. Turn to the next page.
Next page 3	Turn to the next page of your scenario packet.
Continue	I apologize for that inconvenience. Let's continue the survey.
Research assistant help	I am going to ask the research assistant to help you. Just a minute please.
Closing	Thanks. Those are all the questions I have for you. However, the research assistant needs to ask you for additional information and will be with you shortly. Thank you for your time, and enjoy the rest of your day. Goodbye.
