# Supplementary material for: Comprehension and engagement in survey interviews with virtual agents
Source: Front Psychol. 2015 Oct 20;6:1578. doi: 10.3389/fpsyg.2015.01578 (PMC4611966; doi:10.3389/fpsyg.2015.01578)
Supplement: Supplementary file 3 [file Table3.PDF]

Supplementary Table 3 | Online post-interview questionnaire

Please type the scenario packet number.

____

First, we'll ask questions about your experience. You can only respond once for each question, unless it is stated otherwise.

How comfortable were you with Derek at the start of the session?

☐ ☐ ☐ ☐ ☐
Not at all comfortable Very comfortable

As the interview progressed, did your comfort with Derek increase, decrease, or stay the same?

☐ Increase
☐ Stay the same
☐ Decrease

How natural was the interaction with Derek?

☐ ☐ ☐ ☐ ☐
Not at all natural Very natural

How often did Derek seem to act on his own?

☐ ☐ ☐ ☐ ☐
Never All the time

Would you say that Derek acted more like a computer or a person?

☐ Just like a computer
☐ As much like a computer as a person
☐ Just like a person

How much did you enjoy interacting with Derek?

☐ ☐ ☐ ☐ ☐
Did not enjoy at all Thoroughly enjoyed

How frustrating was it to be interviewed by Derek?

☐ ☐ ☐ ☐ ☐
Not at all frustrating Very frustrating

I felt that Derek was…

☐ ☐ ☐ ☐ ☐
Impersonal Personal

☐ ☐ ☐ ☐ ☐
Distant Close

☐ ☐ ☐ ☐ ☐
Inexpressive Expressive

☐ ☐ ☐ ☐ ☐
Insensitive Sensitive

How often did you feel that you were able to answer the questions correctly?

☐ ☐ ☐ ☐ ☐
Never Always

Did you ever ask Derek for clarification?

☐ No: **Why didn't you ask Derek for clarification? Please choose all that apply.**

- ☐ I was too shy.
- ☐ I did not believe Derek was capable of offering clarification.
- ☐ I thought I knew all the correct answers and did not need clarification.
- ☐ I wanted to get the interview over as soon as possible.
- ☐ Other: ______________

☐ Yes: **When you asked Derek for clarification, did you find Derek's reactions…**

☐ ☐ ☐ ☐ ☐
Not at all useful Very useful

☐ ☐ ☐ ☐ ☐
Not at all annoying Very annoying

☐ ☐ ☐ ☐ ☐

Not at all confusing Very confusing

☐ ☐ ☐ ☐ ☐
Not at all frustrating Very frustrating

Did Derek ever provide clarification without you specifically asking for it? [HIGH-DIALOG-CAPABILITY ONLY]

☐ No

☐ Yes:

[IF YES] When Derek provided clarification without your request, did you find this clarification…]HIGH-DIALOG-CAPABILITY ONLY]

☐ ☐ ☐ ☐ ☐
Not at all useful Very useful

☐ ☐ ☐ ☐ ☐
Not at all annoying Very annoying

☐ ☐ ☐ ☐ ☐
Not at all confusing Very confusing

☐ ☐ ☐ ☐ ☐
Not at all frustrating Very frustrating

☐ ☐ ☐ ☐ ☐
Not at all disruptive Very disruptive

If we invited you back to our lab to participate in a similar survey, which of the following would you prefer?

☐ A paper questionnaire
☐ A computerized questionnaire (form)
☐ An interview conducted by Derek
☐ An interview conducted by a human interviewer

You're almost done! Now, we'll ask questions about you. You may respond by typing your answer or making only one selection, unless it is stated otherwise.

What is your sex?

☐ Male ☐ Female

What is your age?

Age: ______

What is your race/ethnicity?

☐ White (non-Hispanic)
☐ Black or African American (non-Hispanic)
☐ Hispanic or Latino
☐ Asian or Pacific Islander
☐ American Indian or Alaskan Native
☐ Multi-racial
☐ Other: ____________________________

Is English your native language?

☐ Yes
☐ No: **Is English the language you currently use most often?**
  ☐ Yes
  ☐ No

What is your highest level of educational attainment?

☐ Some high school or less
☐ High school graduate (including equivalency)
☐ Some college (no degree)
☐ Associate degree
☐ Bachelor's degree
☐ Graduate or professional degree

In which of the following context(s) have you used avatars (that is, animated agents/talking heads)? Please choose all that apply.

☐ Video game consoles
☐ Online computers games
☐ Personal computers games
☐ Instant message
☐ Email

☐ Cell phones
☐ Help assistants (like Clippy from Microsoft Office)
☐ Other: ________________________________
☐ N/A

How often, on average, do you use a computer?

☐ Never (skip next 2 questions—survey complete)
☐ Once a month or less
☐ Once every two weeks
☐ One or two days a week
☐ Three or four days a week
☐ Five to seven days a week

Which of the following computer tasks have you EVER done? Please choose all that apply.

☐ Used email
☐ Visited web sites
☐ Read/posted to blogs
☐ Used chat/instant message programs (VIDEO)
☐ Used chat/instant message programs (AUDIO)
☐ Used chat/instant message programs (TEXT)
☐ Used word processing and/or spreadsheets
☐ Computer programming
☐ Graphic design
☐ Other: (Specify: __________________________________)

Which of the following computer tasks do you do IN A TYPICAL WEEK? Please choose all that apply.

☐ Use email
☐ Visit web sites
☐ Read/post to blogs
☐ Use chat/instant message programs (VIDEO)
☐ Use chat/instant message programs (AUDIO)
☐ Use chat/instant message programs (TEXT)
☐ Use word processing and/or spreadsheets
☐ Computer programming
☐ Graphic design
☐ Other: (Specify: __________________________________)
