# Supplementary material for: Comprehension and engagement in survey interviews with virtual agents
Source: Front Psychol. 2015 Oct 20;6:1578. doi: 10.3389/fpsyg.2015.01578 (PMC4611966; doi:10.3389/fpsyg.2015.01578)
Supplement: Supplementary file 4 [file Table4.PDF]

Supplementary Table 4 | Coding scheme of interviewer and respondent moves.

**Interviewer moves**		
**Code**	**Description**	**Example**
**A**	Reads question	(see Supplementary Table 1)
**B**	Repeats question	(see Supplementary Table 1)
**C**	Administers neutral probe	*Let me repeat the question; whatever it means to you*
**D**	Comments on respondent's confusion	*It looks like you are having some trouble.*
**E**	Offers help/clarification	*What can I help you with*? *Can I help you*?
**F**	Provides full definition related to something respondent said	Full definition of a business (see Supplementary Table 1) when respondent explicitly asks what counts as a business (Employment Question 1)
**G**	Provides partial definition related to something respondent said	Partial definition of a business (see Supplementary Table 2) when respondent explicitly asks what counts as a business
**H**	Provides full definition not directly related to something respondent said	Full definition of a business when respondent explicitly asks what counts as a farm (Employment Question 1)
**I**	Provides partial definition not directly related to something respondent said	Partial definition of a business when respondent explicitly asks what counts as a farm
**J**	Implicitly provides definition by answering respondent's query	*No* when respondent asks if a lamp counts as furniture
**K**	Accepts answer	*Okay*; *Please turn to the next page*
**L**	Section transition	*Now I would like to ask you a few questions about housing*
**M**	Thanks	*Thanks*; *Thank you*.
**N**	Administrative and logistic instrutions	*Turn to the next page; Please wait one moment.*
**O**	Send in research assistant	*I am going to ask the research assistant to help you. Just a minute please.*

**Respondent moves**		
**Code**	**Description**	**Example**
**a**	Correctly answers question	
**b**	Incorrectly answers question	
**c**	Correctly answers with qualification	*I guess 40 hours a week.*
**d**	Incorrectly answers with qualification	
**e**	Reports about circumstances in scenario but does not answer question	*Well, there is a room that was designed as a den but …*
**f**	Asks virtual interviewer to repeat the question.	
**g**	Explicitly requests clarification	*Define full bathroom*; *What do you mean by "furniture"*?
**h**	Expresses confusion about concept (implicitly requesting clarification)	*I don't know whether to count that or not.*
**i**	Back channel utterances	*Okay*; *yes*; *I see*
**j**	Explicitly rejects virtual interviewer's offers of help	*No, I am just reading* after interviewer asks if he can help
**k**	Implicitly rejects virtual interviewer's offers of help	*I am still reading* after interviewer asks if he can help
**l**	Closing	*Goodbye*
**m**	Other	
